# X-Box Binding Protein 1 (XBP1s) Is a Critical Determinant of *Pseudomonas aeruginosa* Homoserine Lactone-Mediated Apoptosis

**DOI:** 10.1371/journal.ppat.1003576

**Published:** 2013-08-22

**Authors:** Cathleen D. Valentine, Marc O. Anderson, Feroz R. Papa, Peter M. Haggie

**Affiliations:** 1 Department of Nephrology, University of California, San Francisco, San Francisco, California, United States of America; 2 Department of Chemistry and Biochemistry, San Francisco State University, San Francisco, California, United States of America; 3 Lung Biology Center, Diabetes Center, and California Institute for Quantitative Biosciences, University of California, San Francisco, San Francisco, California, United States of America; University of Washington, United States of America

## Abstract

*Pseudomonas aeruginosa* infections are associated with high mortality rates and occur in diverse conditions including pneumonias, cystic fibrosis and neutropenia. Quorum sensing, mediated by small molecules including *N*-(3-oxo-dodecanoyl) homoserine lactone (C12), regulates *P. aeruginosa* growth and virulence. In addition, host cell recognition of C12 initiates multiple signalling responses including cell death. To gain insight into mechanisms of C12-mediated cytotoxicity, we studied the role of endoplasmic reticulum stress in host cell responses to C12. Dramatic protection against C12-mediated cell death was observed in cells that do not produce the X-box binding protein 1 transcription factor (XBP1s). The leucine zipper and transcriptional activation motifs of XBP1s were sufficient to restore C12-induced caspase activation in XBP1s-deficient cells, although this polypeptide was not transcriptionally active. The XBP1s polypeptide also regulated caspase activation in cells stimulated with *N*-(3-oxo-tetradecanoyl) homoserine lactone (C14), produced by *Yersinia enterolitica* and *Burkholderia pseudomallei*, and enhanced homoserine lactone-mediated caspase activation in the presence of endogenous XBP1s. In C12-tolerant cells, responses to C12 including phosphorylation of p38 MAPK and eukaryotic initiation factor 2α were conserved, suggesting that C12 cytotoxicity is not heavily dependent on these pathways. In summary, this study reveals a novel and unconventional role for XBP1s in regulating host cell cytotoxic responses to bacterial acyl homoserine lactones.

## Author Summary

Chronic and acute infections associated with *P. aeruginosa* constitute a major healthcare burden. Antimicrobial approaches are currently used against *P. aeruginosa*; however, infections are typically refractory to treatment and drug resistant strains have been isolated. As such, there is urgent need to understand mechanisms of *P. aeruginosa* virulence and for new strategies to fight infections. The *P. aeruginosa*-derived quorum-sensing molecule C12 is recognized by host cells and initiates stress responses including cytotoxicity. In this study, the X-box binding protein 1 transcription factor (XBP1s) was identified as a host factor critical for apoptotic responses initiated by C12 and other similar quorum sensing molecules. Additional C12-initiated host responses, including phosphorylation of p38 MAPK and eIF2α were found to be of lesser importance for C12-initiated cytotoxicity. These studies have broad implications for our understanding of bacterial virulence mechanisms and for development of potential new strategies to combat infections.

## Introduction

*Pseudomonas aeruginosa* is an opportunistic bacterium and infections caused by this pathogen constitute a significant heath-care burden. *P. aeruginosa* is the fourth most-commonly isolated nosocomial pathogen and infections can be fatal, particularly in immuno-compromised subjects such as those with burns or undergoing chemotherapy [1], [2], [3], [4]. Chronic conditions including cystic fibrosis (CF), chronic obstructive pulmonary disease, acquired immunodeficiency syndrome, and non-CF bronchiectasis are also associated with *P. aeruginosa* infection [5], [6], [7]. Antimicrobials are currently used against *P. aeruginosa*; however, infections are typically refractory to therapeutic interventions [8]. Furthermore, drug resistant *P. aeruginosa* strains have been isolated and biofilm formation in chronic conditions enhances antimicrobial resistance and pathogenicity [9], [10]. As such, there is urgent need to understand mechanisms of *P. aeruginosa* virulence and for new strategies to combat infections [10], [11].

**Figure 1:**
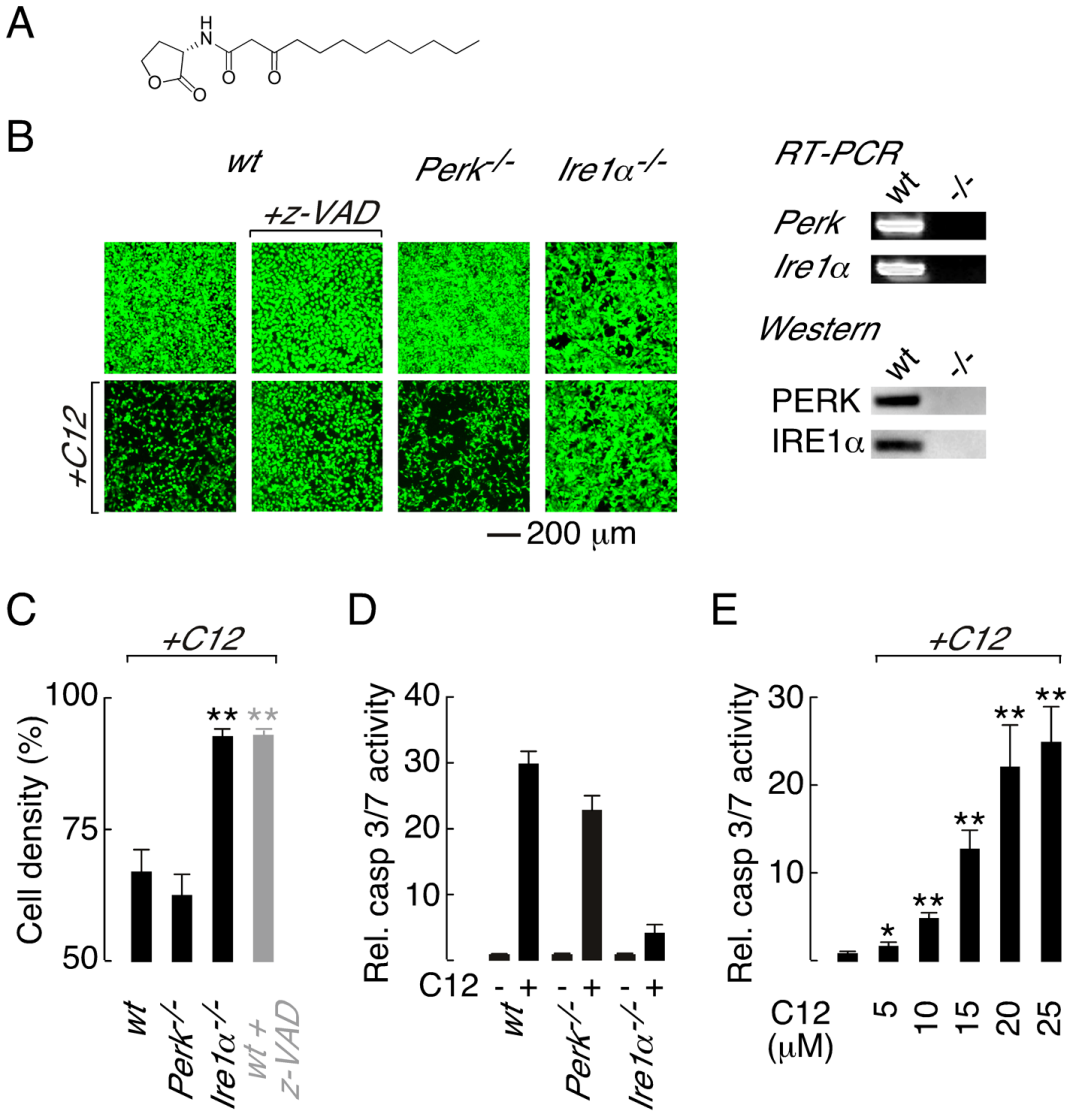
Deletion of *Ire1α* prevents C12 cytotoxicity. A. Structure of the *Pseudomonas aeruginosa N*-(3-oxo-dodecanoyl) homoserine lactone (C12). B. (*left*) Cell density of wt (wild-type; *first panels*), *Perk^−/−^* (*third panels*) and *Ire1α^−/−^* (*fourth panels*) MEFs under control conditions (*top*) and after C12 challenge (25 µM, 4 hours, *bottom*) assessed by calcein AM labelling. Caspase inhibition prevents C12 cytotoxicity as assessed by calcein AM staining of z-VAD-fmk (z-VAD) treated wt MEFs cells (*second panels*). (*right*) RT-PCR (*top*) and western blot (*bottom*) analysis of *Perk^−/−^* and *Ire1α^−/−^* MEFs. C. Quantitative analysis of change in MEF cell density after C12 challenge in wt, *Perk^−/−^* and *Ire1α^−/−^* MEFs and in z-VAD-fmk-treated wt MEFs (*grey bar*). D. Normalized caspase 3/7 activity in wt, *Perk^−/−^* and *Ire1α^−/−^* MEFs in control conditions (−) and after C12 treatment (+; 25 µM, 4 hours). E. Dose response of normalized caspase 3/7 activity in wt MEFs treated with C12 for 4 hours. Statistical analysis was by t-test with reference to data from wt MEFs (C) or control experiments (E); * p<0.005, ** p<0.0001.

The process of quorum sensing (QS) has evolved as a mechanism by which bacterial communities sense cell density and consequently regulate processes such as growth, biofilm formation and expression of virulence factors [12], [13]. Bacteria synthesize small, diffusible molecules to coordinate QS and a major signal generated by *P. aeruginosa* is *N*-(3-oxo-dodecanoyl) homoserine lactone (C12, Figure 1A). In addition to regulating *P. aeruginosa* responses, C12 activates host cell responses via a process termed inter-kingdom signalling [14]. C12 is cytotoxic in a dose dependent manner in macrophages, neutrophils, fibroblasts and epithelial cells [15], [16], [17], [18]. Additional C12-activated host responses include phosphorylation of p38 mitogen activated protein kinase (MAPK), phosphorylation of eukaryotic translation initiation factor 2α (eIF2α) and synthesis of c-*jun* mRNA [17]. C12 represses transcriptional activation of nuclear factor κ-light chain-enhancer of activated B cells (NF-κB) -responsive genes in stimulated macrophages, T-cells and fibroblasts [19], [20], [21]. In contrast, C12 has also been reported to intrinsically activate pro-inflammatory responses, including elevation of prostaglandin E2 and cyclooxygenase 2 levels, in certain cell types [22], [23]. Finally, C12 affects calcium homeostasis by releasing calcium stores from the endoplasmic reticulum (ER) [18], [24], [25].

Knowledge of how host cells recognize and respond to C12 is incomplete, although it is believed that C12 may interact with multiple host targets in a cell-type dependent manner [14]. The bitter taste receptor T2R38 was recently demonstrated to recognize C12 [26]. Stimulation of T2R38 by C12 activates mucociliary clearance and nitric oxide production to facilitate bacterial clearance and killing in primary airway epithelial cultures [26]. Polymorphisms in the T2R38 gene associated with Gram-negative sinonasal infections were demonstrated to reduce airway responsiveness to C12 indicating that C12–host interactions are pathologically relevant [26]. C12 is cell permeable and members of the peroxisome proliferator-activated receptor nuclear hormone receptor family have been proposed to respond to C12 [27]. In contrast, it has been suggested that C12 and other similar acyl homoserine lactones interact directly with host cell membranes to alter membrane polarity and consequently mediate cellular responses [28]. Although innate immune system receptors are capable of recognizing diverse pathogen-derived molecules, Toll-like receptors (TLRs) and the nucleotide-binding, oligomerization domain (NOD)-like receptors Nod1 and Nod2 are not required for C12 recognition [17].

The overall objective of this study was to gain insight into how C12 causes cell death. Several C12-induced cellular responses, including morphological changes to ER structure, phosphorylation of eIF2α and disruption of calcium homeostasis, are characteristic of ER stress [17], [24], [25]. ER stress typically activates intrinsic apoptosis [29], [30] and additional C12-initiated responses including cytochrome *c* release, mitochondrial depolarization and caspase 9 activation, suggest that this pathway is involved in C12 cytotoxicity [17], [18], [25]. As such, we tested the hypothesis that ER stress initiates C12-mediated cell death. Unexpectedly, these studies identified the X box-binding protein 1 transcription factor (XBP1s) as a major regulator of C12-mediated cell death. In this study, we report that: (*i*) XBP1s regulates C12 cytotoxicity by a novel, non-transcriptional mechanism; (*ii*) XBP1s mediates cellular responses to C12 and similar acyl homoserine lactones in a cell type-independent manner and; (*iii*) C12-mediated cellular responses occur via XBP1s-dependent and independent pathways.

## Results

### Deletion of inositol-requiring enzyme 1α is protective against C12-mediated cell death

Three sensors monitor ER protein folding stress; PERK (protein kinase RNA-like ER kinase), IRE1α (inositol-requiring enzyme 1α) and ATF6 (activating transcription factor 6) [30], [31]. Of these sensors, PERK and IRE1α have been associated with ER stress-activated apoptotic responses [31], [32], [33]. Therefore, to investigate the role of ER stress response pathways in C12 cytotoxicity, wild type (wt) murine embryonic fibroblasts (MEFs) and knockout MEFs that do not express PERK (*Perk^−/−^*) or IRE1α (*Ire1α^−/−^*) were challenged with C12 and cytotoxic responses characterized. Significant cell death was observed in C12-treated wt MEFs (∼40% at 4 hours), as assessed by comparison of cell labelling with the supravital dye calcein before and after C12-treatment (Figure 1B, *first panels*, and Figure 1C). The response of *Perk^−/−^* MEFs to C12 was similar to that in wt MEFs indicating that C12 cytotoxicity is not dependent upon PERK activity (Figure 1B, *third panels*, and Figure 1C). In contrast, *Ire1α^−/−^* MEFs were resistant to C12 cytotoxicity suggesting that C12-induced cell death is IRE1α-dependent (Figure 1B, *fourth panels*, and Figure 1C). RT-PCR and western blot analysis was performed to verify the identity of knockout cell lines used in these studies (Figure 1B, *right*).

Caspase (cysteine-dependent aspartate-directed protease) activation has previously been implicated in C12-mediated cell death [17], [18]; however, caspases have not definitively been demonstrated to mediate C12 cytotoxicity. As such, caspase activity in C12-challenged MEFs was characterized to further investigate the mechanism of C12 cytotoxicity. In wt MEFs, the pan-caspase inhibitor z-VAD-fmk inhibited C12-cytotoxicity (∼90% cells remained at 4 hours) suggesting that cell death requires caspase activation (Figure 1B, *second panels* and Figure 1C, *grey bar*). Several families of cysteine proteases can be inhibited by z-VAD-fmk; however, inhibition of cathepsin B (CA-074-Me) or calpain (ALLN) did not reduce C12 toxicity in wt MEFs (Figure S1A). As such, z-VAD-fmk effects in C12-treated MEFs were caspase specific and C12 cytotoxicity is dependent upon caspase activation. Direct measurements of executioner caspase (caspase 3 and 7) activity in MEF cell lines stimulated with 25 µM C12 indicated that *Ire1α* deletion dramatically reduced caspase activation relative to wt and *Perk^−/−^* MEFs (Figure 1D). Reported concentrations of C12 in liquid culture and biofilms vary from 5 to 600 µM [21], [34]; therefore, we assessed caspase activation in wt MEFs in response to low C12 concentrations (Figure 1E and Figure S1B). Executioner caspase activation was significantly enhanced by 4 hour treatment with low C12 concentration (5 µM and 10 µM C12 produced ∼2-fold and ∼5-fold increases respectively) and progressively increased with higher C12 doses. Taken together, these studies indicate that C12-cytotoxicity in MEFs: (*i*) requires caspases such that cell death can formally be classified as apoptosis, (*ii*) is highly dependent on IRE1α activity and, (*iii*) is independent of PERK activity.

### IRE1α deletion indirectly prevents C12 apoptosis

IRE1α is a transmembrane protein that resides in the ER and contains cytoplasmic kinase and endoribonuclease (RNase) activities [30], [35]. During homeostatic conditions, IRE1α RNase activity is limited, although it does mediate sequence specific, non-conventional splicing of a pre-mRNA (*Xbp1u*) to generate mature mRNA (*Xbp1s*) that encodes the X-box binding protein 1 transcription factor (XBP1s) [30], [33], [35]. *Xbp1u* pre-mRNA also encodes XBP1u protein that binds to and regulates XBP1s stability [36]; the internal location of the *Xbp1u* splice site dictates that XBP1u and XBP1s share a common amino terminus. During periods of irremediable ER stress, as observed in pathological conditions such as neurodegeneration and type II diabetes, IRE1α becomes hyper-activated leading to apoptosis driven by promiscuous IRE1α RNase activity and/or activation of apoptosis signal-regulating kinase 1 (ASK1) and its downstream target c-jun NH_2_-terminal kinase (JNK) [33], [37], [38], [39].

**Figure 2:**
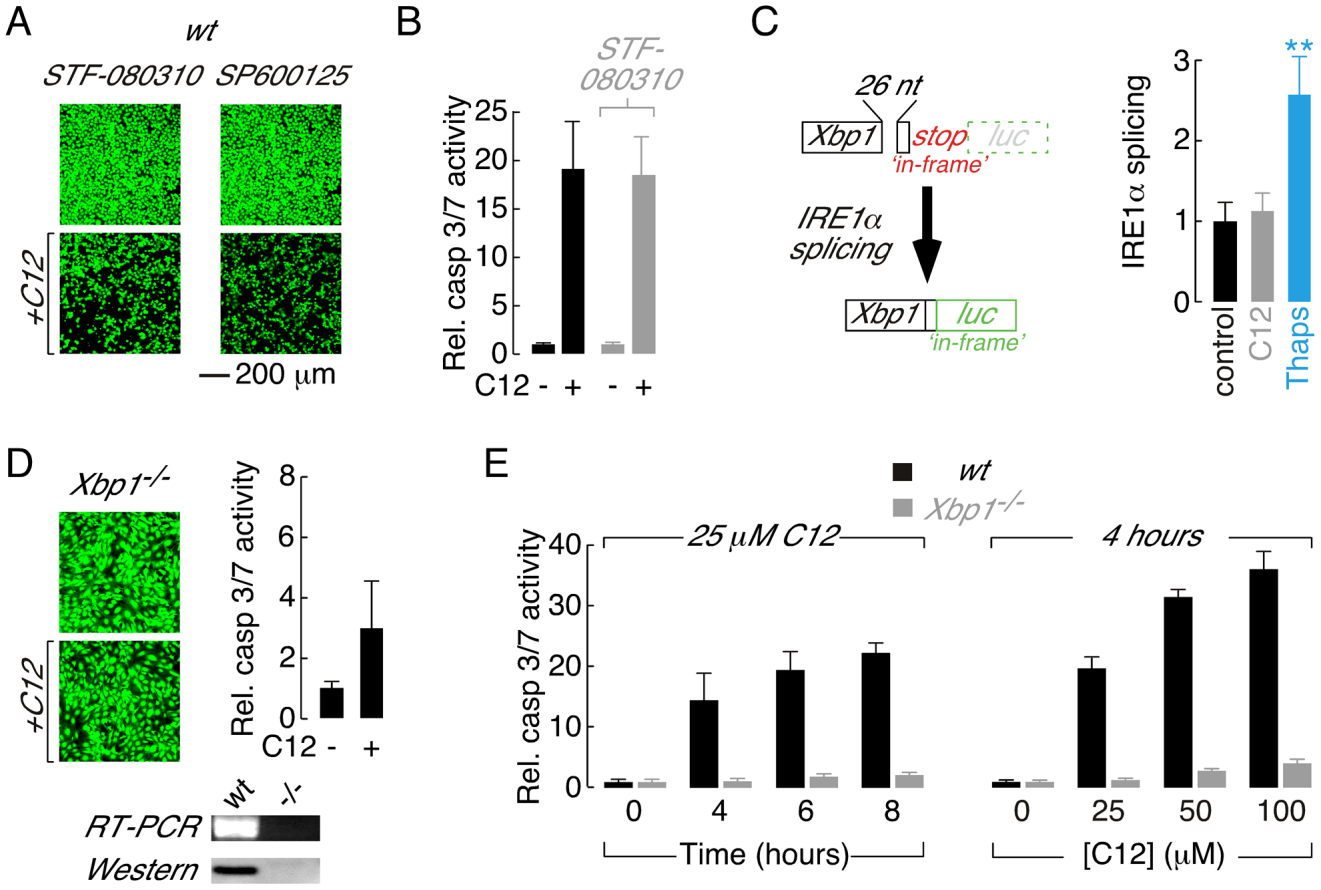
Absence of the XBP1s transcription factor is responsible for reduced C12 cytotoxicity. A. Fluorescence images of calcein AM stained wt MEFs treated with inhibitors of IRE1α RNase activity (STF-080310) and JNK (SP600125) in control conditions (*top*) and with C12 (25 µM, 4 hours, *bottom*). B. Normalized caspase 3/7 activation in wt MEFs in control conditions (*black*) and after treatment with STF-083010 (*grey*). C. Assessment of IRE1α activation in C12-treated cells. (*left*) Schematic of luciferase-based IRE1α activity reporter. Luciferase (*luc*) expression is prevented under control conditions by an ‘*in-frame*’ stop codon (*top*); however, IRE1α activation results in non-conventional splicing and removal of 26 nucleotides (26 nt) from the reporter pre-mRNA which shunts the stop codon ‘*out-of-frame*’ and the luciferase ‘*in-frame*’ (*bottom*). (*right*) IRE1α activation in wt MEFs in control conditions (*black*) and after 2 hours treatment with 25 µM C12 (*grey*) or 250 nM thapsigargin (*blue*). Statistical analysis was by ANOVA with Dunnett post hoc test; ** p<0.0001 versus control cells. D. Deletion of *Xbp1* prevents C12-cytotoxicity assessed by calcein AM labelling (*left*) and caspase 3/7 activation (*right*). (*bottom*) Analysis of *Xbp1^−/−^* MEF by RT-PCR and western blot. E. Analysis of time- (*left*) and dose-dependent (*right*) C12-mediated normalized caspase 3/7 activation in wt (*black*) and *Xbp1^−/−^* (*grey*) MEFs. Cells were treated with 25 µM C12 over 0–8 hours and for 4 hours with 0–100 µM C12. Scale bar in panel A refers to all images.

Treatment of wt MEFs with STF-083010, a selective and irreversible inhibitor of IRE1α RNase activity [39], [40], did not inhibit C12-mediated toxicity or caspase 3/7 activation (Figure 2A, *left* and Figure 2B). Similarly, treatment of wt MEFs with a JNK inhibitor (SP600125) did not reduce C12 cytotoxicity (Figure 2A, *right*) or activation of caspase 3/7 (Figure S1C). A prerequisite for IRE1α-mediated apoptotic signalling is IRE1α activation, a process that involves oligomerization and trans-autophosphorylation [33], [37]. Therefore, to further investigate a possible role of IRE1α in C12-mediated cytotoxic responses, IRE1α activity was directly assessed using a luciferase-based reporter (XBP1-*luc*). The XBP1-*luc* reporter encodes cDNA containing the *Xbp1u* pre-mRNA splice site fused upstream of a luciferase gene ([41], see also MATERIALS AND METHODS). In the absence of IRE1α activity, an ‘*in-frame*’ stop codon prevents luciferase expression (Figure 2C, *top left*); however, upon IRE1α-mediated pre-mRNA splicing (which removes 26 nucleotides), the stop codon is shunted ‘*out-of-frame*’ and luciferase is expressed to report IRE1α activity (Figure 2C, *bottom left*). In wt MEFs expressing XBP1-*luc*, IRE1α activity was not increased above control levels after 2 hours of C12 stimulation (Figure 2C, *right*). In contrast, exposure of cells to thapsigargin, a known ER stress-inducing agent, produced significant enhancement of IRE1α activity over the same time frame (Figure 2C, *right*). Treatment of wt MEFs for 2 hours with C12 or thapsigargin produced relatively little cell loss (<10%, Figure S1A). Data obtained in an additional cell type suggested that the absence of significant IRE1α activation in response to C12 stimulation is a generalized phenomenon (Figure S1D). In summary, these studies: (*i*) provide pharmacological evidence that IRE1α is not directly involved in C12-mediated cytotoxicity and, (*ii*) demonstrate that C12 stimulation of cells does not significantly activate the IRE1α ER stress sensor.

*Ire1α* ^−/−^ MEFs synthesize *Xbp1u* pre-mRNA; however, *Xbp1u* splicing and XBP1s protein are undetectable in this cell type indicating that IRE1α is uniquely responsible for processing of *Xbp1u* pre-mRNA in MEFs ([33], [42], see also Figure S2A). As such, C12 cytotoxic responses were assessed directly in *Xbp1^−/−^* MEFs to test the hypothesis that reduced C12 cytotoxicity in *Ire1α* ^−/−^ MEFs results from loss of XBP1s function(s). C12 stimulation of *Xbp1^−/−^* MEFs produced little apparent reduction in cell density (Figure 2D, *left*) and dramatically reduced activation of executioner caspases relative to wt MEFs (Figure 2D, *right*). The identity of *Xbp1^−/−^* MEFs used in these studies was confirmed by RT-PCR and western blot analysis (Figure 2D, *bottom*). Additional experiments were performed to characterize the apparent tolerance of *Xbp1^−/−^* MEFs to C12 cytotoxicity. Treatment of wt MEFs with C12 produced time- and dose-dependent increases in caspase 3/7 activity, similar to observations made in other cell types [15], [16], [17], [18] (Figure 2E, *black bars*). In contrast, caspase 3/7 activation only increased by ∼2–4-fold in *Xbp1^−/−^* MEFs stimulated with 25 µM C12 for up to 8 hours or with C12 concentrations increasing to 100 µM (Figure 2E, *grey bars*). Similar results were obtained in *Ire1α* ^−/−^ MEFs although control experiments verified that levels of executioner caspases in *Xbp1^−/−^* and *Ire1α^−/−^* MEFs were similar to those in wild type cells (Figure S1E and data not shown). As such, these studies indicate that C12 cytotoxicity requires the presence of XBP1s and that reduced levels of the XBP1s do not merely alter the kinetics or sensitivity of cellular responses to C12.

**Figure 3:**
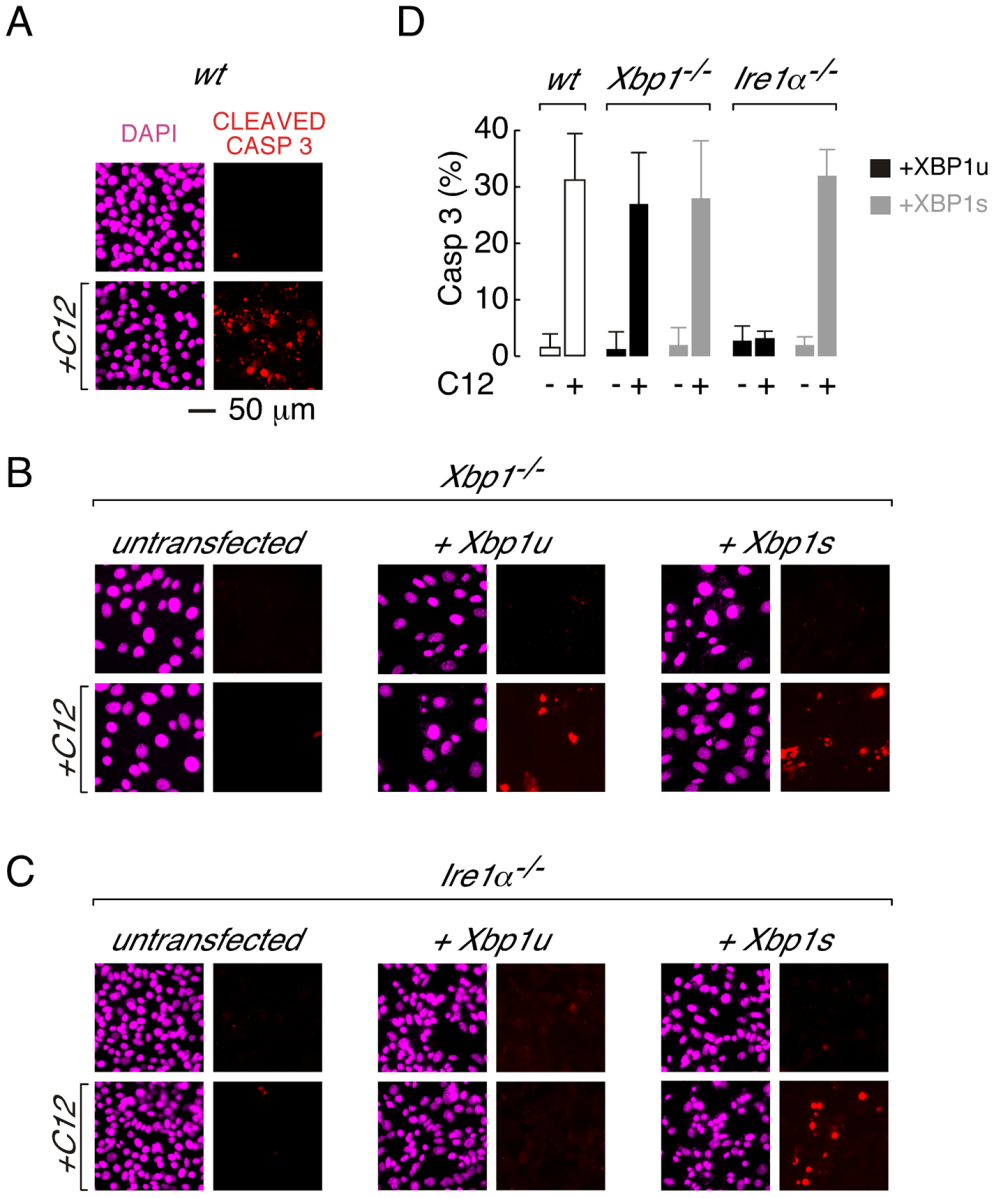
Transient expression of XBP1s restores caspase 3 cleavage in *Xbp1^−/−^* and *Ire1α* ^−/−^ MEFs. A. Caspase activation in C12-treated wt MEFs assessed by cleaved caspase 3 immunocytochemistry. Images are shown in the absence (*top*) and presence (*bottom*) of C12 (25 µM, 2 hours) with DAPI (magenta) and cleaved caspase 3 (red) staining. B. Cleaved caspase 3 immunostaining in C12-treated *Xbp1^−/−^* cells. Untransfected cells (*left panels*) show limited cleaved caspase 3 immunostaining in the presence of C12 although transient expression of cDNA encoding XBP1u (*middle panels*) or XBP1s (*right panels*) restores caspase 3 cleavage upon C12 treatment. C. Cleaved caspase 3 immunostaining in C12-treated *Ire1α^−/−^* cells. Untransfected cells (*left panels*) and cells transfected with cDNA encoding XBP1u (*middle panels*) show limited cleaved caspase 3 immunostaining in the presence of C12 although expression of cDNA encoding XBP1s (*right panels*) restores caspase 3 cleavage upon C12 treatment. D. Number of cells showing cleaved caspase 3 immunostaining after C12 treatments. Data is shown for wt MEFs (*white bars*) and *Xbp1^−/−^* and *Ire1α^−/−^* MEFs transiently transfected with cDNA encoding XBP1u (*black*) or XBP1s (*grey*) in the presence and absence of C12 (25 µM, 2 hours). Scale bar in panel A refers to all images.

To investigate whether XBP1s loss-of-function was directly responsible for C12 tolerance, *Xbp1^−/−^* and *Ire1α* ^−/−^ MEFs were transiently transfected with *Xbp1* cDNA and responses to C12 were characterized. As predicted from direct measurement of executioner caspase activation, treatment of wt MEFs with C12 produced robust caspase 3-cleavage that could be detected by immunostaining (Figure 3A). Similarly, as expected from measurements of executioner caspase activity, cleaved caspase 3 was essentially absent in C12-stimulated *Xbp1^−/−^* and *Ire1α* ^−/−^ MEFs (Figure 3B and C, *left panels*). Transfection of *Xbp1* ^−/−^ MEFs with cDNA encoding either *Xbp1u* pre-mRNA (which can be processed by IRE1α; Figure 3B, *middle*) or *Xbp1s* mRNA (Figure 3B, *right*) restored caspase 3 cleavage upon C12 treatment in a large number of cells. In contrast, transfection of *Ire1α* ^−/−^ MEFs with *Xbp1s* cDNA (Figure 3C *right*), but not *Xbp1u* cDNA (which is present endogenously but remains unspliced, Figure 3C, *middle*), restored caspase-3 cleavage upon C12 treatment. In control experiments, C12-treatment of *Xbp1* ^−/−^ MEFs transfected with an empty plasmid was not associated with caspase 3-cleavage, verifying that transfection of this cell type was not responsible for caspase activation (Figure S1F). Taken together, these experiments indicate that expression of XBP1s in XBP1s-deficient MEFs (*Xbp1^−/−^* or *Ire1α* ^−/−^ MEFs) is sufficient to re-establish C12-mediated caspase cleavage.

### C12-mediated apoptosis does not require XBP1s transcriptional activity

**Figure 4:**
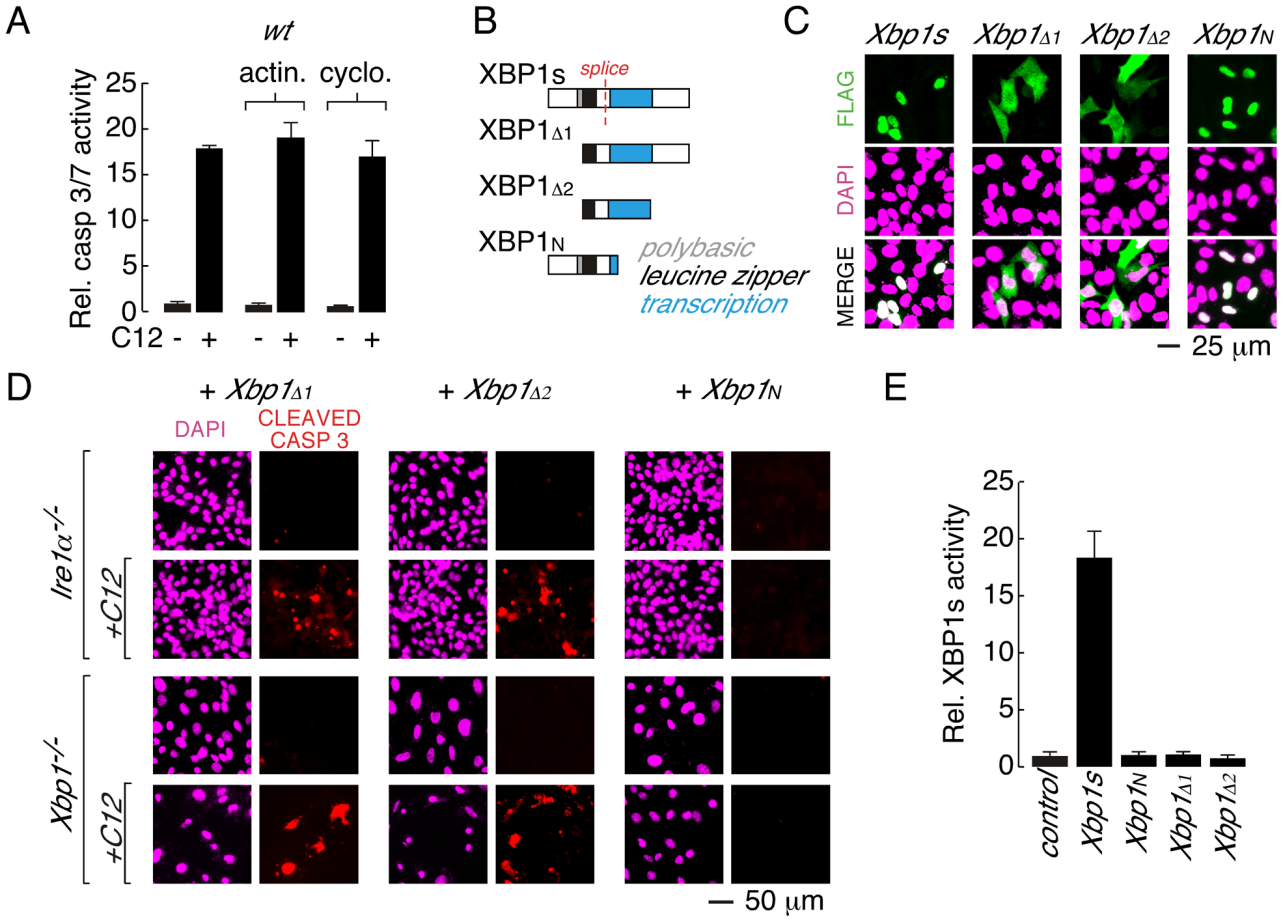
Leucine zipper and transcriptional activation domains of XBP1s are sufficient for C12-mediated caspase activation. A. Acute inhibition of RNA or protein synthesis with actinomycin (actin.) or cycloheximide (cyclo.) does not inhibit C12-induced caspase activation in wt MEFs. B. Domain structure of XBP1s and XBP1s truncations. XBP1s contains a polybasic domain (*grey*), leucine zipper domain (*black*) and transcriptional activation domain (*blue*). The location of the splice site that converts XBP1u pre-mRNA to XBP1s mRNA is also shown (*red*). The FLAG-tag on all constructs is located at the amino-terminus. C. Analysis of cellular localization of XBP1s and XBP1s truncations by immunocytochemistry. Panels are shown for anti-FLAG immunostaining (*top*), DAPI staining (*middle*), and merged images (*bottom*). D. Expression of XBP1s truncations that contain the leucine zipper and transcriptional activation domains (XBP1_Δ1_ and XBP1_Δ2_) is sufficient to restore C12-mediated caspase activation in *Ire1α* ^−/−^ (*top panels*) and *Xbp1^−/−^* (*bottom panels*) MEFs. Expression of XBP1_N_ failed to restore C12-mediated caspase activation. Images are shown for DAPI staining (magenta) and caspase immunostaining (red) in the absence and presence of C12. E. XBP1s truncations that restore C12-mediated caspase activation (XBP1_Δ1_ and XBP1_Δ2_) are transcriptionally inactive. Normalized XBP1s transcriptional activity was measured in wt MEFs using an XBP1s-responsive luciferase-based reporter and co-expression of control plasmid, XBP1s, or XBP1 truncations. Individual scale bars are shown for panels C and D.

XBP1s is a member of the cAMP response element-binding protein (CREB)/ATF basic region–leucine zipper family of transcription factors and, consistent with this function, XBP1s is modular containing a polybasic region, a leucine zipper motif and a transcriptional activation domain [36] (Figure 4B). Therefore, experiments were conducted to investigate whether XBP1s transcriptional activity was required for C12-mediated apoptosis. Treatment of wt MEFs with inhibitors of transcription (actinomycin) or translation (cycloheximide) during C12 stimulation did not prevent caspase activation (Figure 4A). As such, in response to C12-stimulation, neither acute activation of XBP1s transcriptional activity nor acute protein synthesis of an XBP1s-dependent transcript are responsible for C12-induced apoptosis.

To gain insight into the role of XBP1s domains in C12-mediated apoptosis, a series of XBP1s truncation constructs were generated (Figure 4B). In wt MEFs, XBP1s is predominantly localized to the nucleus, although in agreement with prior biochemical analysis, low levels of XBP1s could also be detected in the cytoplasm [36] (Figure 4C and Figure S2B). Deletion of the amino terminus and polybasic region from XBP1s produced a truncation construct (XBP1_Δ1_) that was distributed throughout the cell (Figure 4B and 4C). When expressed in *Ire1α* ^−/−^ or *Xbp1^−/−^* MEFs, XBP1_Δ1_ was able to re-establish caspase 3 cleavage upon C12 treatment (Figure 4D, *left*). In a similar manner, an XBP1s truncation construct that consisted of only the leucine zipper and transcriptional activation domains (XBP1_Δ2_) was distributed throughout the cell (Figure 4B and 4C) and restored caspase 3 cleavage in C12-stimulated *Ire1α* ^−/−^ and *Xbp1^−/−^* MEFs (Figure 4D, *middle*). A truncation construct encompassing the amino-terminal domains of XBP1s (XBP1_N_, Figure 4B) failed to re-establish caspase 3 cleavage in C12 stimulated *Ire1α* ^−/−^ or *Xbp1^−/−^* MEFs (Figure 4D, *right*). As such, consideration of C12-mediated caspase 3-cleavage in XBP1s-deficient MEFs expressing XBP1_Δ2_ or XBP1_N_ suggested that the transcriptional activation domain of XBP1s contains a protein sequence that is responsible for C12-mediated caspase activation. However, instability of additional XBP1s truncation constructs that were generated precluded further analysis to identify a minimal XBP1s region that is necessary for C12-mediated caspase 3-cleavage. For instance, truncation constructs such as those that encode the XBP1s transcriptional activation domain and carboxy-terminus or the XBP1s transcriptional activation domain alone could not be detected by immunocytochemistry or western blot analysis (Figure S2C and data not shown). As such, our analysis indicates that the leucine zipper and transcriptional activation domains of XBP1s (XBP1_Δ2_) constitute a minimal XBP1s region that can be stably expressed and is sufficient for caspase activation in C12-treated *Ire1α* ^−/−^ or *Xbp1^−/−^* MEFs.

Next, experiments were performed to assess the ability of XBP1s truncation constructs to mediate transcriptional activity. In these assays, wt MEFs were co-transfected with an XBP1s-responsive luciferase reporter and either XBP1s or an XBP1s truncation construct [43], [44]. As expected, robust transcriptional responses were observed when XBP1s was co-expressed with the reporter (Figure 4E). In contrast, XBP1_N_, which lacks a transcriptional activation domain, was not associated with transcriptional activity (Figure 4E). The XBP1_Δ1_ and XBP1_Δ2_ truncation constructs that restore caspase 3-cleavage in C12-treated *Ire1α* ^−/−^ and *Xbp1^−/−^* MEFs were also not associated with XBP1s-dependent transcriptional activity (Figure 4E and Figure S2A). Taken together, these studies indicate that: (*i*) the leucine zipper and transcriptional activation domains of XBP1s (XBP1_Δ2_) constitute a minimal, stable XBP1s fragment that restores caspase activation upon C12 stimulation in XBP1s-deficient cells and; (*ii*) C12-mediated apoptosis does not require XBP1s-transcriptional activity.

### XBP1s regulates a common mechanism for acyl homoserine lactone-mediated caspase activation

**Figure 5:**
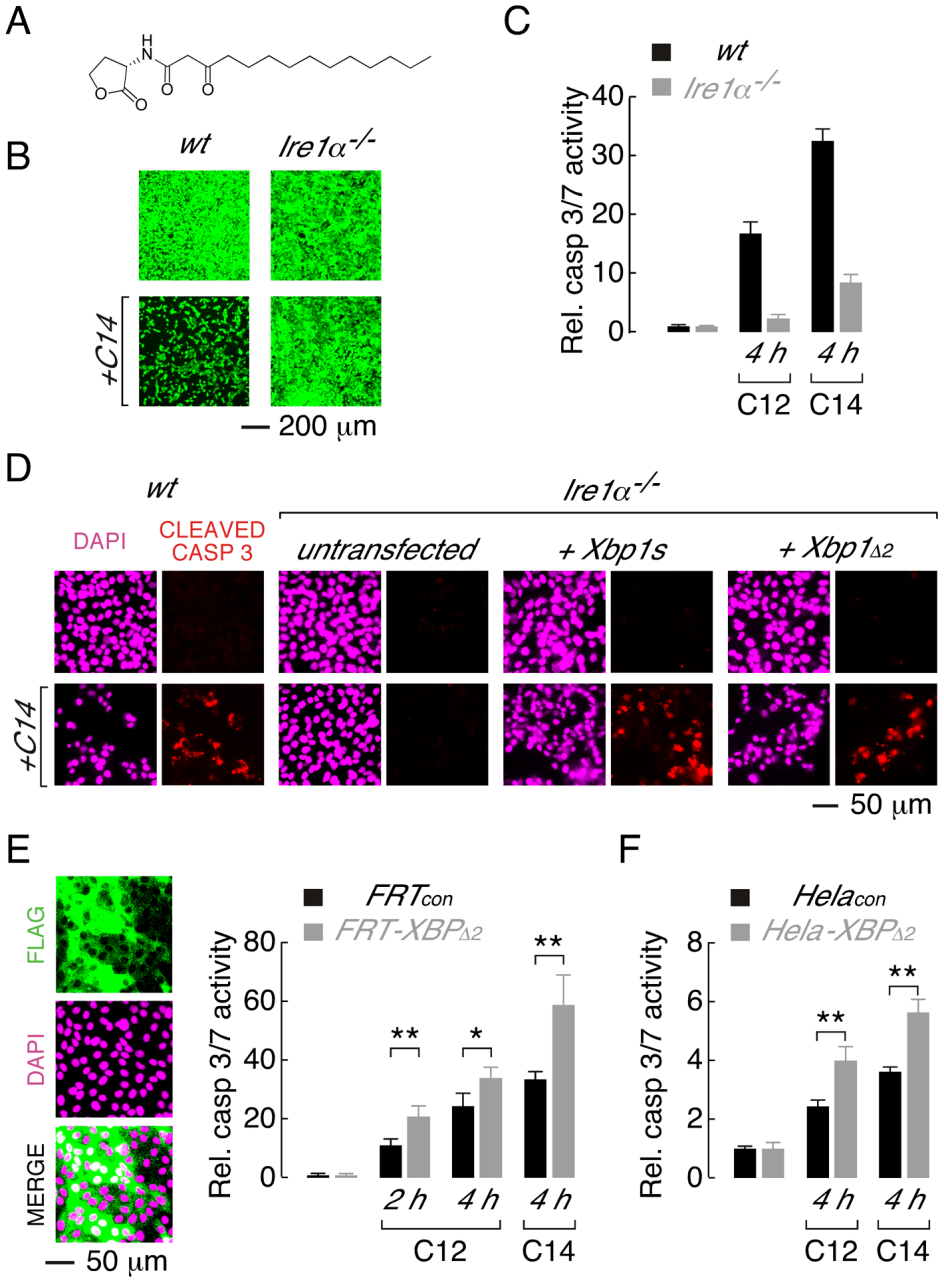
XBP1s mediates cellular responses to the C14 homoserine lactone and in different cell types. A. Structure of *N*-(3-oxo-tetradecanoyl) homoserine lactone (C14). B. *Ire1α* ^−/−^ MEFs are protected from C14-mediate cell death as assessed by calcein AM staining. C. Normalized caspase 3/7 activity in wt (*black*) and *Ire1α^−/−^* (*grey*) MEFs in control conditions and after treatment with C12 or C14 (25 µM, 4 h). D. Caspase activation in C14-treated MEFs assessed by cleaved caspase 3 immunocytochemistry. Images are shown in the absence (*top*) and presence (*bottom*) of C14 (25 µM, 2 hours) with DAPI (magenta) and cleaved caspase 3 staining (red). Wildtype MEFs (*first panels*) and *Ire1α^−/−^* MEFs that are either untransfected (*second panels*) or transfected to express XBP1s (*third panels*) or XBP1_Δ2_ (*fourth panels*) are shown. E. Over-expression of XBP1_Δ2_ enhances executioner caspase activation when heterologously expressed in FRT cells. (*left*) Fluorescence images of cell enriched for expression of XBP1_Δ2_ (FRT-XBP1_Δ2_), anti-FLAG immunostaining (*top*), DAPI fluorescence (*middle*) and a merged image (*bottom*) are shown. (*right*) Caspase 3/7 activation is enhanced in FRT-XBP1_Δ2_ cells (*grey bars*) relative to control cells (*black bars*), by C12 (25 µM) at 2 and 4 hours and by C14 (25 µM) at 4 hours. F. Over-expression of XBP1_Δ2_ enhances C12- and C14- (25 µM) mediated caspase 3/7 activation when heterologously expressed in Hela cells. Statistical analysis was by t-test and for pairwise comparison of control and XBP1_Δ2_ overexpressing cells, * p<0.001 and ** p<0.0001.

Acyl homoserine lactones are a common chemical scaffold used by diverse bacterial species for QS [12]. Therefore, experiments were performed to investigate if XBP1s-dependent mechanisms mediate cellular responses to *N*-(3-oxo-tetradecanoyl) homoserine lactone (C14, Figure 5A), a QS molecule generated by the gram-negative, pathogenic bacteria *Yersinia enterolitica* and *Burkholderia pseudomallei*. Treatment of wt MEFs with C14 produced significant cell death, as assessed by calcein AM fluorescence (Figure 5B, *left*), and activation of executioner caspases (Figure 5C, *right*). In contrast, *Ire1α^−/−^* MEFs were largely resistant to C14-mediated cytotoxicity (Figure 5B, *right*) and demonstrated significantly reduced caspase activation (∼20%) relative to wt cells (Figure 5C, *right*). As expected from direct measurements of caspase activity, robust cleavage of caspase 3 could be detected in C14-treated wt MEFs; however, cleaved caspase 3 immunostaining was largely absent in *Ire1α* ^−/−^ MEFs (Figure 5D). Transfection of *Ire1α* ^−/−^ MEF cells to express either XBP1s or XBP1_Δ2_ restored caspase 3-cleavage in C14 treated cells (Figure 5D), as observed in C12-treated cells.

Studies were also performed to investigate whether XBP1_Δ2_ affects cytotoxic response to acyl homoserine lactones in other cell types. A Fisher rat thyroid (FRT) cell line was generated that was highly enriched for expression of XBP1_Δ2_ (FRT-XBP1_Δ2_, Figure 5E, *left*). Treatment of FRT-XBP1_Δ2_ with C12 or C14 resulted in increased activation of executioner caspases relative to control cells (FRT_con_, Figure 5E, *right*). Similarly, enhanced caspase activation was observed in a Hela cell line enriched for XBP1_Δ2_ expression upon treatment with C12 or C14 (Figure 5F). It is noteworthy that in all cell types studied C14 produced higher levels of caspase activation than C12. Studies with further acyl homoserine lactones indicated that acyl chains of >12 carbon atoms and substitution at the third carbon of the acyl chain are required for toxicity (Figure S3). Taken together, these experiments indicate that: (*i*) the leucine zipper and transcriptional activation domains (XBP1_Δ2_) of XBP1s regulate activation of caspases in response to different homoserine lactones and, (*ii*) XBP1_Δ2_ results in increased caspase activation in the presence of endogenous XBP1s.

### C12 activates alternative cellular responses via XBP1s-independent mechanisms

**Figure 6:**
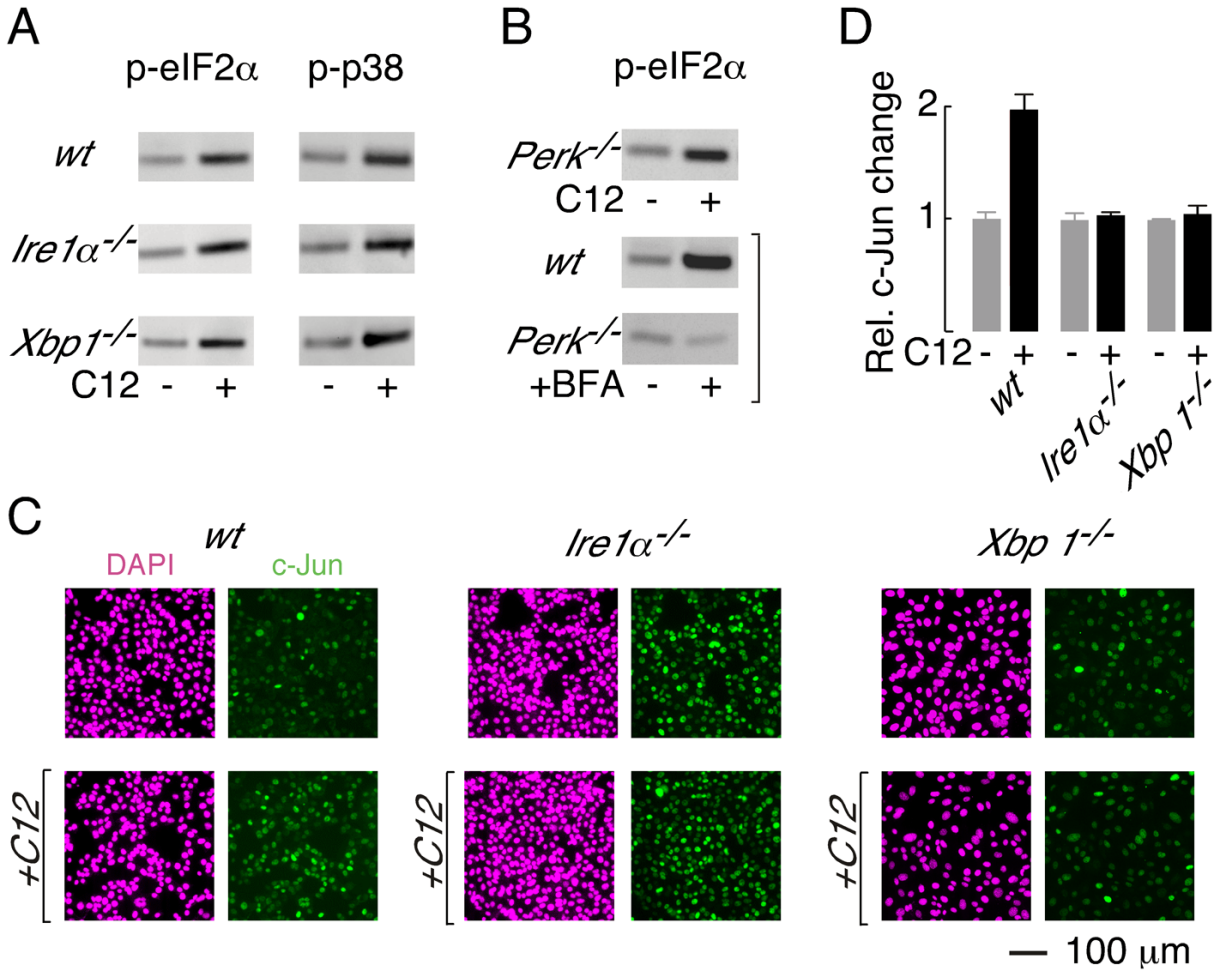
XBP1-independent cellular responses to C12. A. Immunoblot analysis of phosphorylated eIF2α (p-eIF2α, *left*) and phosphorylated p38 (p-p38, *right*) levels in wt, *Ire1α* ^−/−^ and *Xbp1^−/−^* MEFs. Cells were treated with 25 µM C12 for 45 minutes. B. (*top*) C12 treatment (25 µM, 45 min) of *Perk^−/−^* MEFs results in phosphorylation of eIF2α (*bottom*) Control experiments confirm that ER stress (brefeldin A (BFA), 2 hours) in wt MEFs, but not *Perk^−/−^* MEFs, produces p-eIF2α. C. C12 treatment increases c-Jun levels in wt MEFs but not in *Ire1α* ^−/−^, and *Xbp1^−/−^* MEFs. Cells were treated with 25 µM C12 for 90 minutes (*bottom*) and c-Jun levels were characterized by image analysis of immunostained cells (green). Images of DAPI staining are also shown (magenta). D. Quantitative analysis of normalized c-Jun levels in wt, *Ire1α* ^−/−^, and *Xbp1^−/−^* MEFs. Scale bar in panel C refers to all images.

C12 activates multiple stress response signalling pathways in host cells [14], [17]. The observation that *Ire1α* ^−/−^ and *Xbp1^−/−^* MEFs are protected from C12-induced cell death afforded the opportunity to investigate whether distinct cellular signalling responses are associated with or unrelated to C12 cytotoxicity. Phosphorylation of eIF2α and p38 MAPK have previously been reported in primary macrophages, fibroblasts and epithelial cells [17]. In agreement with this study, increased phospho-eIF2α (p-eIF2α; ∼2.5-fold change) and phospho-p38 MAPK (p-p38 MAPK; ∼2.5-fold change) could also be detected in wt MEFs after C12 exposure for 45 minutes (Figure 6A, *top panels*). A similar pattern of phosphorylation of eIF2α and p38 MAPK was observed in C12-treated *Ire1α* ^−/−^ and *Xbp1^−/−^* MEFs (Figure 6A, *middle and bottom panels*; ∼2.5 and ∼3-fold increases in p-eIF2α, and ∼2.5 and ∼3.5-fold increases in p-p38 MAPK); indicating that these responses are independent of XBP1s and IRE1α activity. As both *Ire1α* ^−/−^ and *Xbp1^−/−^* MEFs are resistant to C12 cytotoxicity, these studies also suggest that phosphorylation of p-eIF2α and p-p38 MAPK does not contribute greatly to C12-mediated cell death in MEFs. Consistent with this notion, inhibition of p38 MAPK activity (using SB203580 or SB202190) did not inhibit C12 cytotoxicity or caspase 3/7 activation in wt MEFs (data not shown).

PERK phosphorylates eIF2α during periods of ER stress [32]; therefore, experiments were performed to investigate whether PERK is activated by C12. Phosphorylation of eIF2α was, however, preserved in C12-treated *Perk^−/−^* MEFs (Figure 6B, *top*; ∼3-fold increase). As expected, control experiments confirmed that ER stress induced by brefeldin A (BFA) produced p-eIF2α in wt MEFs (∼4-fold increase) but not *Perk^−/−^* MEFs (∼0.9-fold change) (Figure 6B, *middle and bottom*). Together, these studies indicate that C12 challenge activates alternative kinase(s) activity to generate p-eIF2α; however, they do not formally demonstrate that PERK does not contribute to eIF2α phosphorylation.

Levels of c-*jun* mRNA have also been reported to increase in C12-treated primary macrophages, fibroblasts and epithelial cells [17]. In wt MEFs, c-Jun protein levels increased by approximately two-fold after 90 minutes treatment with 25 µM C12 (Figure 6C, *left*, and Figure 6D). In contrast, c-Jun protein levels were not altered by C12 in *Ire1α* ^−/−^ or *Xbp1^−/−^* MEFs (Figure 6C, *middle and right*, and Figure 6D). At present, these studies cannot distinguish whether c-Jun levels increase in wt MEFs in an XBP1s (or IRE1α) -dependent manner or due to C12-mediated cytotoxicity; however, these studies confirm that C12 cytotoxicity is associated with increased c-Jun levels. It is noteworthy that in unstimulated conditions, c-Jun levels in wt and knockout MEFs varied by approximately three-fold; as such, elevated levels of c-Jun, at least in the absence of XBP1s, are not sufficient to mediate C12 cytotoxicity. These studies indicate that C12-mediated host cell responses occur via both XBP1s-dependent and -independent mechanisms and C12-mediated production of p-eIF2α and p-p38 MAPK in MEFs does not contribute greatly to cell death.

## Discussion

**Figure 7:**
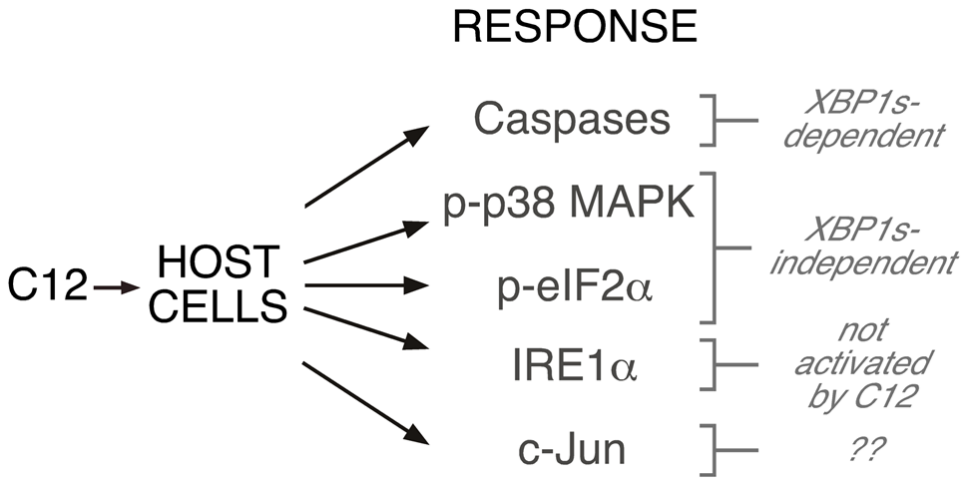
Role of XBP1s in C12-mediated cellular responses. Activation of caspases is dependent upon XBP1s, albeit by a mechanism that does not involve transcription. Phosphorylation of p38 MAPK and eIF2α is XBP1s-independent. Stimulation of cells with C12 does not activate IRE1α and presumably does not activate other ER stress response pathways. No inferences about c-Jun synthesis can be drawn from the current study.

Infections associated with *P. aeruginosa* result in significant mortality and constitute a major worldwide healthcare burden. Our understanding of *P. aeruginosa* virulence mechanisms is limited and there is an urgent need to identify new approaches that can be employed to reduce *P. aeruginosa* infectivity. *P. aeruginosa* employ QS to regulate growth, virulence factor expression and biofilm formation. In addition to quorum signalling, *P. aeruginosa*-derived C12 homoserine lactone activates several host cell responses including cytotoxicity. The major conclusion of this study is that C12-mediated host cell cytotoxic responses largely require expression of the XBP1s transcription factor (Figure 7). Cell death and caspase activation were dramatically reduced (∼95%) in cells that cannot generate XBP1s (*Ire1α* ^−/−^ MEFs) or lack the XBP1 gene (*Xbp1^−/−^* MEFs). To the best of our knowledge, this study represents the first description of cells that are C12-tolerant due to genetic ablation of a protein. Restoring XBP1s expression in either *Ire1α* ^−/−^ or *Xbp1^−/−^* MEFs was sufficient to re-establish caspase 3 cleavage upon C12 stimulation. Although the present study does not provide full mechanistic detail, we demonstrate that a fragment of XBP1s encompassing the leucine zipper and transcriptional activation domains (XBP1_Δ2_) that is not transcriptionally active is sufficient to restore C12-mediated caspase 3 cleavage. Knockout cell lines were used to incisively demonstrate the role of XBP1s in homoserine lactone-mediated cell death; however, expression of XBP1_Δ2_ in rat and human epithelial cells resulted in enhanced caspase activation in response to C12- and C14-stimulation, indicating that XBP1s can mediate apoptotic responses in distinct cell types. Further experiments will be required to establish that XBP1s regulates C12-mediated apoptosis in cells that are exposed to *P. aeruginosa*, including macrophages, neutrophils and airway epithelial cells; however, XBP1s is expressed at high levels in these cells [45].

The concentration of C12 observed in liquid cultures is ∼5 µM and biofilms may generate up to ∼600 µM C12 [21], [34]. Caspase activation and the appearance of characteristic apoptotic morphology were observed in wt MEFs that were treated with 5 µM C12. Cellular responses, including phosphorylation of p38 MAPK and eIF2α were reported in cells exposed to 5–10 µM C12 [17]. *In vivo* studies indicate that low concentrations of C12 (1–10 µM) produce profound responses [46]. Furthermore, it has been proposed that host cells may be exposed to high local C12 concentrations generated by *P. aeruginosa* structures including micro-colonies and biofilms [47], [48]. The only rigorous validated C12 receptor is T2R38, this bitter taste receptor is activated by C12 to stimulate host antimicrobial responses including nitric oxide synthesis and mucociliary clearance [26]. In human subjects, common loss-of-function polymorphism in T2R38 were linked to host susceptibility to sinonasal gram-negative bacterial infections, suggesting that T2R38 is a key determinant of upper airway innate defense mechanisms [26]. In primary cell cultures, TR238-mediated antimicrobial responses were stimulated by C12 in the concentration range 10–100 µM. As such, we predict that C12 concentrations considered in these studies will be clinically relevant. Formal verification that C12-mediated cytotoxicity enhances *P. aeruginosa* virulence will require identification of pharmacological tools that can be used to block caspase activation *in vivo*.

XBP1s is a member of the CREB/ATF basic region-leucine zipper family of transcription factors and performs pleiotropic transcriptional functions. Approaches including expression profiling and chromatin immunoprecipitation have revealed that XBP1s regulates gene expression for both constitutive and inducible arms of ER stress response pathways; however, XBP1s-responsive promoter regions also regulate expression of genes involved in signal transduction, redox homeostasis, cell growth and differentiation and carbohydrate metabolism [35], [44], [49]. Studies in knock-out mice have revealed that XBP1s is essential for hepatogenesis, B lymphocytes differentiation, cardiac myogenesis and development of exocrine tissue [50], [51], [52], [53]. Targeted deletion of XBP1s in murine intestinal epithelial cells results in enhanced inflammatory responses and *Xbp1* polymorphisms are associated with Crohn's disease and ulcerative colitis [54]. In *C. elegans*, *P. aeruginosa*-mediated PMK-1 p38 MAPK activation during development induces ER stress that is lethal in the absence of XBP1s [55]. In general, these studies indicate that XBP1s typically acts in a protective manner to restore cellular homeostasis when protein load on the ER is excessive [35]. XBP1s function has, however, been linked to cell death and over long time courses (days) increased levels of XBP1s have been associated with apoptosis in endothelial and pancreatic β cells [56], [57]. The current study represents the first descriptions of an unconventional (non-transcriptional) role for XBP1s and of a function for XBP1s in regulating acute apoptosis.

Additional insights into the role of cell stress response pathways in C12 stimulated cells are afforded by the present study (summarized in Figure 7). Activation of ER stress response pathways has been proposed as a mechanism by which C12 initiates host cell responses [14], [48]. Direct assessment of IRE1α splicing activity and pharmacological inhibition of apoptotic pathways that are initiated by IRE1α suggests that C12 does not activate IRE1α. An additional inference that can be drawn from these observations is that ‘*house-keeping*’ levels of XBP1s are sufficient to mediate apoptotic responses to C12. However, it is noteworthy that immune responses initiated by *P. aeruginosa* (via TLR4 and TLR5) will result in IRE1α-activation and increased levels of XBP1s [58]. ER stress-induced PERK activation has also been proposed to be responsible for C12-mediated eIF2α phosphorylation; however, robust eIF2α phosphorylation was observed in *Perk^−/−^* MEFs suggesting that an alternative kinase(s) catalyzes this response. Although the present studies do not formally rule out PERK-mediated eIF2α phosphorylation, this scenario seems unlikely given that: (*i*) C12-mediated IRE1α ER stress responses are negligible, (*ii*) PERK and IRE1α sense ER stress by a similar mechanism [59] and, (*iii*) deletion of PERK does not alter cytotoxic responses to C12. In response to cell stress, eIF2α is also phosphorylated by eIF2α kinase 1 (the heme-regulated inhibitor kinase), eIF2α kinase 2 (protein kinase RNA-activated, PKR) or eIF2α kinase 4 (GCN2) [60]; further work will be required to establish which eIF2α kinase(s) responds to C12. The role of individual stress response pathways to overall C12-mediated cytotoxicity is not well understood. The observations that p38 MAPK phosphorylation is conserved in *Ire1α* ^−/−^ and *Xbp1^−/−^* MEFs and that pharmacological inhibition of p38 MAPK does not prevent C12-mediated apoptosis suggest that this pathway is of limited importance to C12-mediated cell death. Similarly, eIF2α phosphorylation is preserved in *Ire1α* ^−/−^ and *Xbp1^−/−^* MEFs also suggesting that this cellular response has little relevance to C12-mediated apoptosis. Finally, it is noteworthy that caspases are activated in *Ire1α* ^−/−^ and *Xbp1^−/−^* MEFs in response to C12-stimulation, albeit at dramatically reduced levels (∼5%) relative to wt MEFs. As such, our study indicates that XBP1s-dependent and -independent processes that contribute to caspase activation are initiated in C12-stimulated cells, but that XBP1s-dependent mechanisms are dominant.

How might XBP1s contribute to cell death in C12-stimulated cells? Our study formally verifies that caspase activation mediates apoptotic cell death in MEFs. Executioner caspase activity was ostensibly used as a metric of cell death; however, caspase 8 was also activated in C12-stimulated MEFs (data not shown) as previously reported in other cell types [16], [18]. Caspase 8 activation typically indicates that extrinsic apoptotic pathways initiated by cell surface receptors are operating [29]. As such, we speculate that XBP1s promotes initiation of extrinsic apoptotic responses through formation of the death-inducing signalling complex in a C12-dependent manner. Precedents of leucine zipper-containing proteins causing intrinsic and extrinsic apoptosis have been reported. Similar to XBP1s, the prostrate apoptosis response-4 protein (Par-4) contains a leucine zipper motif and nuclear localization sequences. Depending upon cell type, Par-4 has been demonstrated to either sensitize cellular responses to apoptotic stimuli or to directly activate apoptosis; however, the mechanism of these responses is not fully elucidated [61]. Further studies will be required to test the hypothesis that XBP1s mediates activation of extrinsic apoptotic responses in a C12-dependent fashion.

In summary, the present study identifies XBP1s as a key determinant of apoptotic responses initiated by QS molecule generated by *Pseudomonas aeruginosa*, *Yersinia enterolitica* and *Burkholderia pseudomallei*. The leucine zipper and transcriptional activation domains of XBP1 are sufficient to mediate apoptotic responses; however, XBP1s transcriptional activity is not required for cell death. These studies advance our understanding of host responses to C12 and should facilitate rational design of approaches to investigate whether C12-mediated host cell apoptosis is pathologically relevant.

## Materials and Methods

### Cell lines and cell culture

Immortalized wt and knockout MEF cell lines used in this study were previously described and RT-PCR analysis of these cells was performed using published primer sequences [32], [33], [37], [39]. MEFs were cultured in DMEM H21 supplemented with 10% fetal bovine serum, 2 mM glutamine, 100 U/ml penicillin, 100 µg/ml streptomycin and nonessential amino acids. Fischer rat thyroid (FRT) epithelial cells were obtained from the UCSF cell culture facility and cultured in Ham's F12 supplemented as for MEFs. Hela cells were obtained from the UCSF cell culture facility and cultured in DMEM H21 supplemented as for MEFs. MEF and FRT cells were transfected using jetPRIME (Polyplus Transfection) and Hela cells were transfected with Lipofectamine LTX with Plus reagent (Life Technologies).

### Reagents and chemicals

Homoserine lactones (including C12 (*N*-(3-oxododecanoyl)-l-homoserine lactone) and C14 (*N*-(3-oxodotetradecanoyl)-l-homoserine lactone)) were obtained from Sigma. For some experiments, C12 was prepared using a previously described synthetic route [62]. Structure and purity (>99%) of synthesized C12 were confirmed by proton NMR, and caspase activation in wt or *Xbp1* ^−/−^ MEFs was similar in response to synthesized or purchased C12. Details of homoserine lactone dosing is provided in Figure Legends or text. Calcein acetoxymethyl ester (AM), DAPI and thapsigargin was obtained from Life Technologies and used as directed by the manufacturer. Pharmacological modulators were purchased from various sources (as detailed below) and cells were typically pre-treated with compounds for 30 minutes, at concentrations given in parenthesis, prior to homoserine lactone challenge or other experimental maneuvers. Cycloheximide (5 µg/ml) and CA-074-Me (10 µM) were obtained from Calbiochem. STF-080310 (50 µM) was obtained from Tocris. Z-VAD-fmk (10 µM) was obtained from Promega. SB203580 (10 µM), SB202190 (10 µM), SP600125 (10 µM), ALLN (10 µM), brefeldin A (1.25 µg/ml) and actinomycin (1 µg/ml) were obtained from Santa Cruz Biotechnology.

### Antibodies, western blotting and immunocytochemistry

Antibodies against, IRE1α, PERK, cleaved and full length caspase 3 and 7, p-eIF2α, p-p38 MAPK and c-Jun were obtained from Cell Signalling. Antibody against XBP1 was purchased from Santa Cruz. Antibody against FLAG-tag was obtained from Sigma. Western blotting was performed using standard procedures with chemiluminescence detection. Immunocytochemistry was performed on fixed and permeabilized cells using indirect immunodetection with appropriate fluorophore-conjugated secondary antibodies and DAPI as a counterstain. Imaging was performed using a Nikon Eclipse TE2000U inverted microscope equipped with an Exfo X-Cite light source, Hamamatsu EM-CCD deep-cooled camera and appropriate filter cubes and objectives (Nikon S Fluor 20× N.A. 0.75 or Plan Fluor 40× N.A. 0.75). For quantitative immunodetection, cell from different experiments were labelled in parallel, imaged with identical microscope settings and analyzed using Fiji image processing software.

### Apoptosis assays and cell density analysis

Caspase activity was measured using Caspase-Glo homogeneous luminescent assays (Promega). In different experiments, caspase activation in C12-treated wt MEFs varied by ∼50% for undetermined reasons. As such, control experiments were always performed and are presented, such that control data and experimental maneuvers can be directly compared. Normalized caspase activation data is presented as mean ± S.D. for an individual experiment (comprised of 6–12 individual measurements), and is representative of at least duplicate experiments. To characterize cell density, cells were labelled with calcein-AM and imaged using a Nikon Eclipse TE2000U inverted microscope (described above) equipped with a Nikon Plan Fluor 10×/NA 0.3 objective. Relative cell number was calculated from background-corrected, integrated fluorescence areas and cell density was derived by comparison of relative cell number before and after an experimental maneuver. Data is presented as mean ± S.E. and was derived from 3–5 independent experiments in which 3–6 cell areas were imaged per experiment.

### Plasmids, molecular biology and luciferase-based reporter assays

Plasmids containing FLAG-tagged murine XBP1u and XBP1s cDNA were obtained from Addgene (plasmids 21832 and 21833; [63]). For experiments, the coding regions for XBP1u and XBP1s were excised as *Hin*dIII/*Xba*I fragments, subcloned into pcDNA3.1/Hygro, and an oligo duplex was used to regenerate the FLAG-tag (amino acid sequence MDYKDDDDL). Carboxy-terminal FLAG-XBP1s truncations (Figure 4B) were generated by pcr as *Hin*dIII/*Xba*I-tagged amplicons that were subcloned into pcDNA3.1/Hygro containing an in-frame FLAG-tag. The amino-terminal FLAG-XBP1s truncation (Figure 4B) was generated by pcr as a *Bgl*II/*Eco*RI-tagged amplicon that was subcloned into the *Bam*HI/*Eco*RI sites of pcDNA3.1; for experiments, FLAG-tagged XBP1 amino terminus was subsequently subcloned into pcDNA3.1/Hygro as an *Nhe*I/*Xba*I fragment. Domain boundaries for truncations were based on those previously reported for human XBP1s [36]. For FLAG-XBP1s, the polybasic, leucine zipper (which contains amino acids involved in transcription factor dimerization) and transcriptional activation domains start at Arg76, Ala94 and Ala168, the transcriptional activation domain ends at Val283 and the amino terminal domain ends at Iso197 (corresponding to residues Arg68, Ala86, Ala160, Val275 and Iso189 of untagged XBP1s). Sequence analysis was used to confirm all constructs generated in this study. For experiments in MEFs that assessed the role of full length and truncated XBP1 regions in caspase activation, pcDNA3.1/Hygro was used as a control (empty) vector. Cell lines enriched for expression of an XBP1s deletion construct (XBP1_Δ2_, Figure 4B) were generated by repeated culture of transfected cells in 300 µg/ml hygromycin; cells transfected with pcDNA3.1/Hygro and selected in 300 µg/ml hygromycin (FRT_con_ and Hela_con_) were used as a control.

A luciferase-based reporter of IRE1α activity was generously provided by Albert Koong (Stanford University). Briefly, this reporter (termed XBP1-*luc*) consists of cDNA encoding the first 208 amino acids of human *Xbp1*, including a 26-nucleotide splice site that is recognized by IRE1α, fused to firefly luciferase [41]. A plasmid-based vector was generated for the current studies by subcloning reporter cDNA (provided in the pLPCX retroviral vector, Clontech) into pcDNA3.1/Hygro as a *Hin*dIII/*Not*I fragment. Assays of IRE1α splicing activity were performed in transiently transfected wt MEFs or in an FRT cell population enriched for expression of the reporter construct by repeated culture of cells in 300 µg/ml hygromycin. A luciferase based reporter of XBP1s activity was purchased (pGL4[*lu2P*/ATF6-RE/Hygro] (Promega)); this reporter contains five direct repeats of the sequence 5′-ATCGAGACAGGTGCTGACGTGGCATTC-3′ and is similar to a previously described XBP1s-responsive reporter (UPRE/5×ATF6GL3) [43], [44]. Assays of XBP1 transcriptional activity were performed in MEFs transiently transfected with plasmids expressing full length or truncated XBP1s (or pcDNA3.1/Hygro as a control) and pGL4[*lu2P*/ATF6-RE/Hygro] at a ratio of 9∶1. Luciferase-based reporter assays were performed using the Bright-Glo Assay system as directed by the manufacturer (Promega).

## Supporting Information

Figure S1 A. Cell density assessment using calcein AM labelling and fluorescence microscopy. (*column 1*) Representative image of control cells. (*column 2*) Inhibition of proteolytic enzymes by CA-074-Me (10 µM) and ALLN (10 µM) does not prevent C12-mediated cell death (25 µM, 4 h), confirming that z-FAD-fmk inhibits caspases (see also Figure 1). (*column 3*) CA-074-Me and ALLN are not associated with toxicity over the time course of experiments. (*column 4*) Treatment of cells with C12 (25 µM) or thapsigargin (250 nM) for 2 hours produces limited cell loss (see also Figure 2C). B. Calcein AM labelling of wt MEFs treated with low concentrations of C12 for 4 hours. Examples of cells with clear apoptotic morphology are highlighted with arrowheads (see also Figure 1E). C. Inhibition of JNK does not inhibit caspase activation (see also Figure 2A). D. (*left*) Relative IRE1α activity in FRT cells measured using XBP1-*luc* (see also Figure 2C). Cells were treated with the ER stress inducing agents tunicamycin (tun, 0.5 µg/ml) and thapsigargin (thaps, 250 nM) or C12 (25 µM) for 4 hours. (*right*) Normalized caspase 3/7 activation in FRT cells treated as in IRE1α activity measurements. C12 did not significantly increase IRE1α activity relative to control levels although C12-stimulation resulted in robust caspase 3/7 activation. ER stress inducing agents did not induce caspase 3/7 activation over the duration of experiments. Statistical analysis was by ANOVA with Dunnett post hoc test using untreated cells as the control condition; ** p<0.0001. E. Western blot of (uncleaved) caspase 3 and caspase 7 in wt, *Ire1α^−/−^* and *Xbp1^−/−^* MEFs. F. Transfection of *Xbp1^−/−^* MEFs with a control (empty) plasmids did not result in caspase 3 cleavage upon C12 treatment (25 µM, 2 h). Staining is shown for DAPI (*left*) and cleaved caspase 3 (*right*) (see also Figure 3).
(TIF)

Figure S2 A. Measurement of XBP1s-mediated transcriptional activity in wt, *Ire1α^−/−^* and *Xbp1^−/−^* MEFs (see also Figure 4E). In all cell types, *Xbp1s* co-transfection with the XBP1s-responsive reporter construct produced robust transcriptional activity. Co-transfection of wt and *Xbp1^−/−^* MEFs with *Xbp1u* cDNA produced limited transcriptional activity, consistent with the ability of these cells to process *Xbp1u* pre-mRNA and generate XBP1s via IRE1α activity (as expected, intrinsic activity of IRE1α is limited such that XBP1s-transcriptional activity in *Xbp1u* transfected cells is lower than that in *Xbp1s* transfected cells). In contrast, *Ire1α^−/−^* MEFs were unable to process *Xbp1u* cDNA such that no transcriptional activity was reported. No transcriptional activity was reported in any cell type for co-transfection with plasmids encoding XBP1_N_ and XBP_Δ2_. Statistical analysis was by ANOVA with Dunnett post hoc test; * p<0.05 and ** p<0.0001 versus control data. (*inset*) Anti-FLAG-tag western blot of wt MEFs expressing XBP1s truncation constructs to confirm appropriate expression. B. Immunostaining of FLAG-XBP1s transfected wt MEFs. Camera sensitivity was adjusted such that low levels of XBP1s could be detected in the cytoplasm of certain transfected cells (see also Figure 4C). C. Schematic representation of XBP1s truncation constructs that were generated, confirmed by sequence analysis and failed to express in wt MEFs. XBP1s contains a polybasic domain (*grey*), leucine zipper domain (*black*; which consists of the amino acids involved in transcription factor dimerization) and transcriptional activation domain (*blue*). The location of the splice site that converts XBP1u pre-mRNA to XBP1s mRNA is also shown (*red*). Leu-to-ala mutants of XBP1s and XBP_Δ2_ contained alanine residues in place of leucine residues at all seven positions in the leucine zipper motif and were generated using gBlocks Gene Fragments (Integrated DNA Technologies).
(TIF)

Figure S3 A. Structures of acyl homoserine lactones used in these studies: C6 (*N*-(ß-Ketocaproyl)-l-homoserine lactone); C10 (*N*-(3-Oxodecanoyl)-l-homoserine lactone); C12; C14; C14-b (*N*-Tetradecanoyl-dl-homoserine lactone); C14-OH (*N*-(3-Hydroxytetradecanoyl)-dl-homoserine lactone). Carbons at position 1 and 3 are highlighted in red (*top*). B. Representative images of calcein AM labelled wt MEFs treated with acyl homoserine lactones (4 h, 25 µM or 50 µM of racemic mixtures; see also Figs. 1 and 5). C. Caspase 3/7 activation in wt (*left*) and *Ire1α^−/−^* (*right*) MEFs after treatment with acyl homoserine lactones (4 h, 25 µM or 50 µM of racemic mixtures). No activation of caspases was observed when acyl chains were <12 carbon atoms in length. Substitutions at carbons one and three are required for caspase activation and 3-oxo- substitutions produce significantly greater activation of caspases than 3-hydroyx- substitutions. *Ire1α^−/−^* MEFs are protected from C12, C14 and C14-OH caspase activation relative to wt MEFs.
(TIF)
