# Correction: X-Box Binding Protein 1 (XBP1s) Is a Critical Determinant of *Pseudomonas aeruginosa* Homoserine Lactone-Mediated Apoptosis

**DOI:** 10.1371/journal.ppat.1005628

**Published:** 2016-05-18

**Authors:** Cathleen D. Valentine, Marc O. Anderson, Feroz R. Papa, Peter M. Haggie

The authors would like to correct Fig 2. During composition of this figure, images presented in Panel A for cell treatment with STF-080310 (*left panels*, *top and bottom*) were inadvertently selected from a dataset conducted using SP600125 (as depicted in Fig 2A, *right panels*). As such, incorrect images are shown for cell treatment with STF-080310, and duplication of images for control data occurred. The corrected version of Fig 2 contains appropriate images for cells treated with STF-080310, although no other changes were made to the figure.

**Fig 2:**
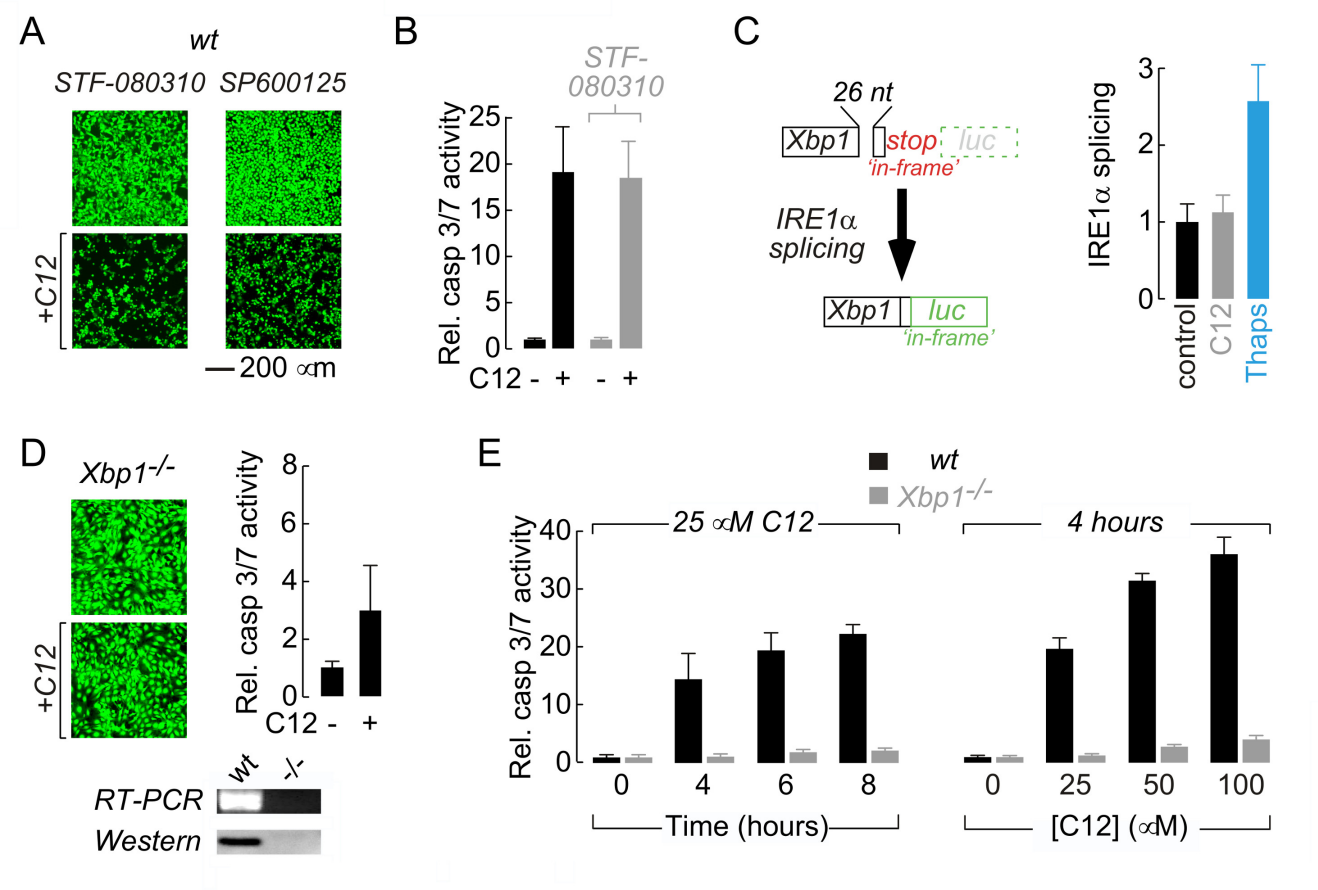
Absence of the XBP1s transcription factor is responsible for reduced C12 cytotoxicity. A. Fluorescence images of calcein AM stained wt MEFs treated with inhibitors of IRE1α RNase activity (STF-080310) and JNK (SP600125) in control conditions (*top*) and with C12 (25 μM, 4 hours, *bottom*). B. Normalized caspase 3/7 activation in wt MEFs in control conditions (*black*) and after treatment with STF-083010 (*grey*). C. Assessment of IRE1α activation in C12-treated cells. (*left*) Schematic of luciferase-based IRE1α activity reporter. Luciferase (*luc*) expression is prevented under control conditions by an ‘*in-frame*’ stop codon (*top*); however, IRE1α activation results in non-conventional splicing and removal of 26 nucleotides (26 nt) from the reporter pre-mRNA which shunts the stop codon ‘*out-of-frame*’ and the luciferase ‘*in-frame*’ (*bottom*). (*right*) IRE1α activation in wt MEFs in control conditions (*black*) and after 2 hours treatment with 25 μM C12 (*grey*) or 250 nM thapsigargin (*blue*). Statistical analysis was by ANOVA with Dunnett post hoc test; ** p<0.0001 versus control cells. D. Deletion of *Xbp1* prevents C12-cytotoxicity assessed by calcein AM labelling (*left*) and caspase 3/7 activation (*right*). (*bottom*) Analysis of *Xbp1−/−* MEF by RT-PCR and western blot. E. Analysis of time- (*left*) and dose-dependent (*right*) C12-mediated normalized caspase 3/7 activation in wt (*black*) and *Xbp1−/−* (*grey*) MEFs. Cells were treated with 25 μM C12 over 0–8 hours and for 4 hours with 0–100 μM C12. Scale bar in panel A refers to all images.

The authors confirm that these changes do not alter their findings. The authors have provided raw images of the new data presented in Fig 2A as Supporting Information (S1 Fig). No alterations need be made to the Fig 2 legend.

## Supporting Information

S1 Fig Original Image data used in Fig 2 for cells treated with STF-080310. Images were acquired as described in the Materials and Methods Section (using a Nikon TE2000 microscope equipped with a 16-bit Hamamatsu EM-CCD). To generate colorized images in Fig 2A, full-field images were binned (2×2), converted to 8-bit, colorized using a green lookup table, and brightness / contrast were adjusted to generate images that could be readily interpreted / visualized when presented in their final, published form. All image manipulations used Fiji and procedures were applied equally to all pixels in an image. To generate this Supporting Figure,.tif files were opened in Fiji, copied (using ‘Copy to System’ on an Apple computer), pasted into a Microsoft PowerPoint file, and saved as a PDF.
(PDF)
