# Supplementary figures and images for: X-Box Binding Protein 1 (XBP1s) Is a Critical Determinant of Pseudomonas aeruginosa Homoserine Lactone-Mediated Apoptosis
Source: PLoS Pathog. 2013 Aug 22;9(8):e1003576. doi: 10.1371/journal.ppat.1003576 (PMC3749957; doi:10.1371/journal.ppat.1003576)

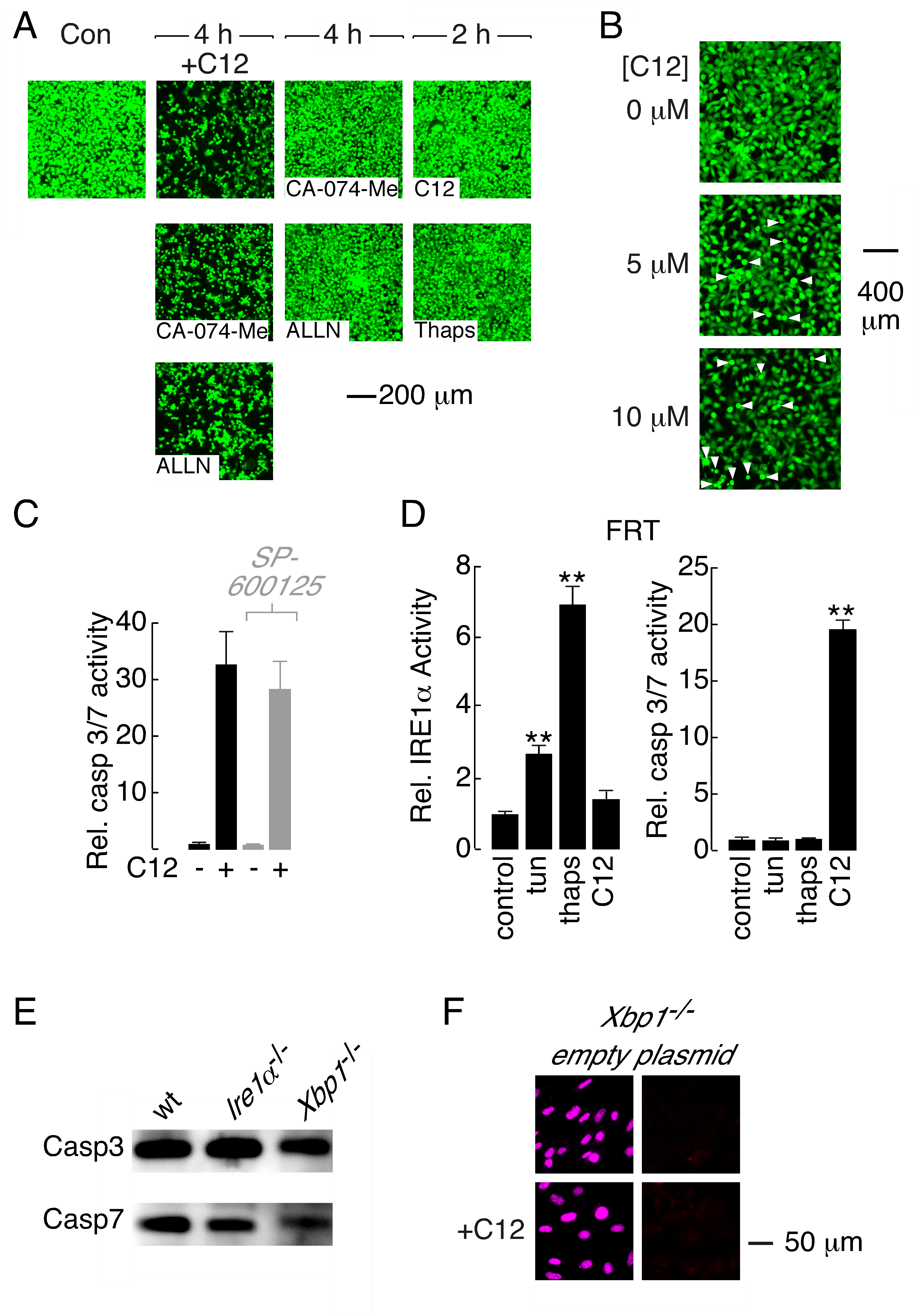

Supplement: Figure S1 — A. Cell density assessment using calcein AM labelling and fluorescence microscopy. (column 1) Representative image of control cells. (column 2) Inhibition of proteolytic enzymes by CA-074-Me (10 µM) and ALLN (10 µM) does not prevent C12-mediated cell death (25 µM, 4 h), confirming that z-FAD-fmk inhibits caspases (see also Figure 1). (column 3) CA-074-Me and ALLN are not associated with toxicity over the time course of experiments. (column 4) Treatment of cells with C12 (25 µM) or thapsigargin (250 nM) for 2 hours produces limited cell loss (see also Figure 2C). B. Calcein AM labelling of wt MEFs treated with low concentrations of C12 for 4 hours. Examples of cells with clear apoptotic morphology are highlighted with arrowheads (see also Figure 1E). C. Inhibition of JNK does not inhibit caspase activation (see also Figure 2A). D. (left) Relative IRE1α activity in FRT cells measured using XBP1-luc (see also Figure 2C). Cells were treated with the ER stress inducing agents tunicamycin (tun, 0.5 µg/ml) and thapsigargin (thaps, 250 nM) or C12 (25 µM) for 4 hours. (right) Normalized caspase 3/7 activation in FRT cells treated as in IRE1α activity measurements. C12 did not significantly increase IRE1α activity relative to control levels although C12-stimulation resulted in robust caspase 3/7 activation. ER stress inducing agents did not induce caspase 3/7 activation over the duration of experiments. Statistical analysis was by ANOVA with Dunnett post hoc test using untreated cells as the control condition; ** p<0.0001. E. Western blot of (uncleaved) caspase 3 and caspase 7 in wt, Ire1α−/− and Xbp1−/− MEFs. F. Transfection of Xbp1−/− MEFs with a control (empty) plasmids did not result in caspase 3 cleavage upon C12 treatment (25 µM, 2 h). Staining is shown for DAPI (left) and cleaved caspase 3 (right) (see also Figure 3). (TIF) [file ppat.1003576.s001.tif]

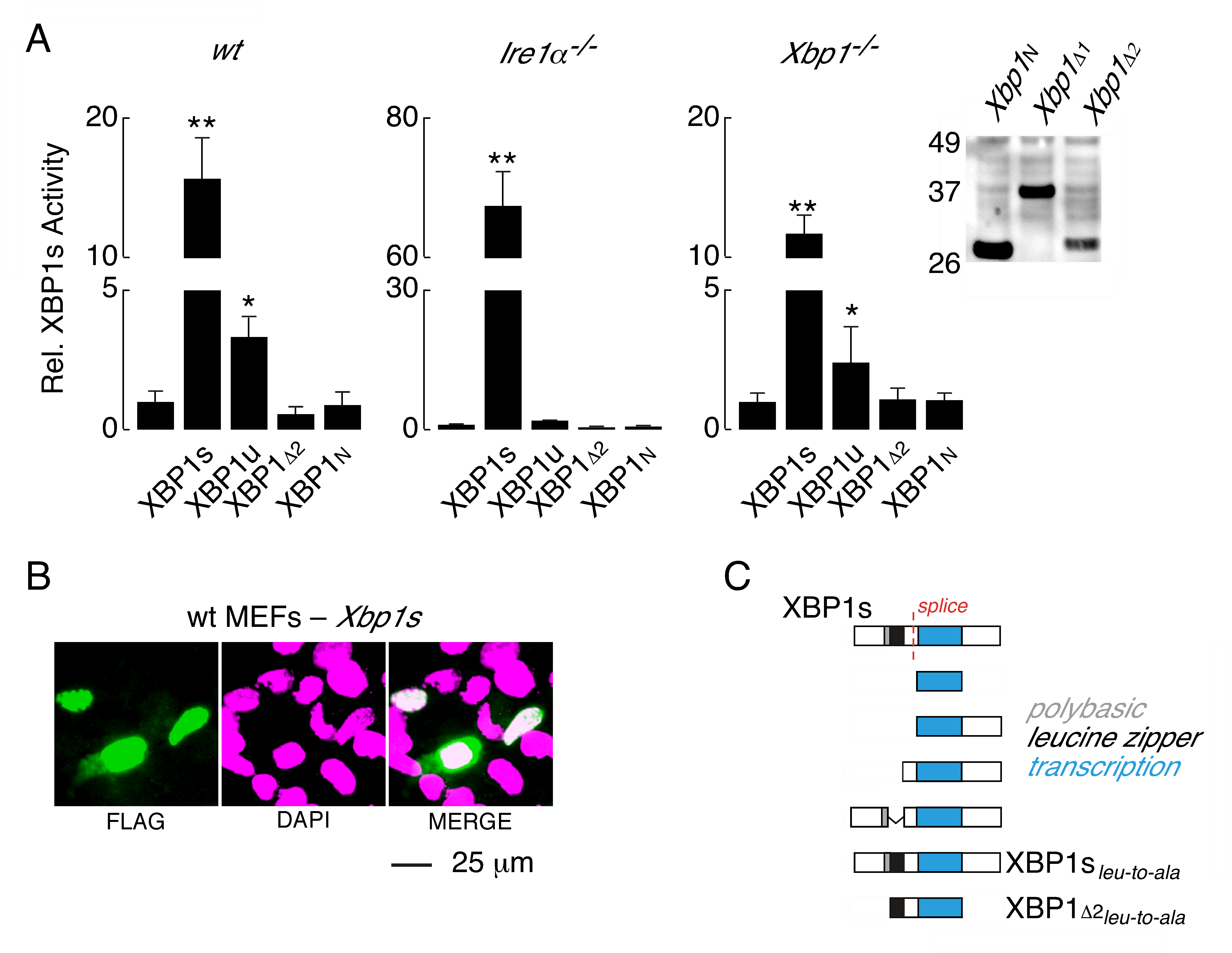

Supplement: Figure S2 — A. Measurement of XBP1s-mediated transcriptional activity in wt, Ire1α−/− and Xbp1−/− MEFs (see also Figure 4E). In all cell types, Xbp1s co-transfection with the XBP1s-responsive reporter construct produced robust transcriptional activity. Co-transfection of wt and Xbp1−/− MEFs with Xbp1u cDNA produced limited transcriptional activity, consistent with the ability of these cells to process Xbp1u pre-mRNA and generate XBP1s via IRE1α activity (as expected, intrinsic activity of IRE1α is limited such that XBP1s-transcriptional activity in Xbp1u transfected cells is lower than that in Xbp1s transfected cells). In contrast, Ire1α−/− MEFs were unable to process Xbp1u cDNA such that no transcriptional activity was reported. No transcriptional activity was reported in any cell type for co-transfection with plasmids encoding XBP1N and XBPΔ2. Statistical analysis was by ANOVA with Dunnett post hoc test; * p<0.05 and ** p<0.0001 versus control data. (inset) Anti-FLAG-tag western blot of wt MEFs expressing XBP1s truncation constructs to confirm appropriate expression. B. Immunostaining of FLAG-XBP1s transfected wt MEFs. Camera sensitivity was adjusted such that low levels of XBP1s could be detected in the cytoplasm of certain transfected cells (see also Figure 4C). C. Schematic representation of XBP1s truncation constructs that were generated, confirmed by sequence analysis and failed to express in wt MEFs. XBP1s contains a polybasic domain (grey), leucine zipper domain (black; which consists of the amino acids involved in transcription factor dimerization) and transcriptional activation domain (blue). The location of the splice site that converts XBP1u pre-mRNA to XBP1s mRNA is also shown (red). Leu-to-ala mutants of XBP1s and XBPΔ2 contained alanine residues in place of leucine residues at all seven positions in the leucine zipper motif and were generated using gBlocks Gene Fragments (Integrated DNA Technologies). (TIF) [file ppat.1003576.s002.tif]

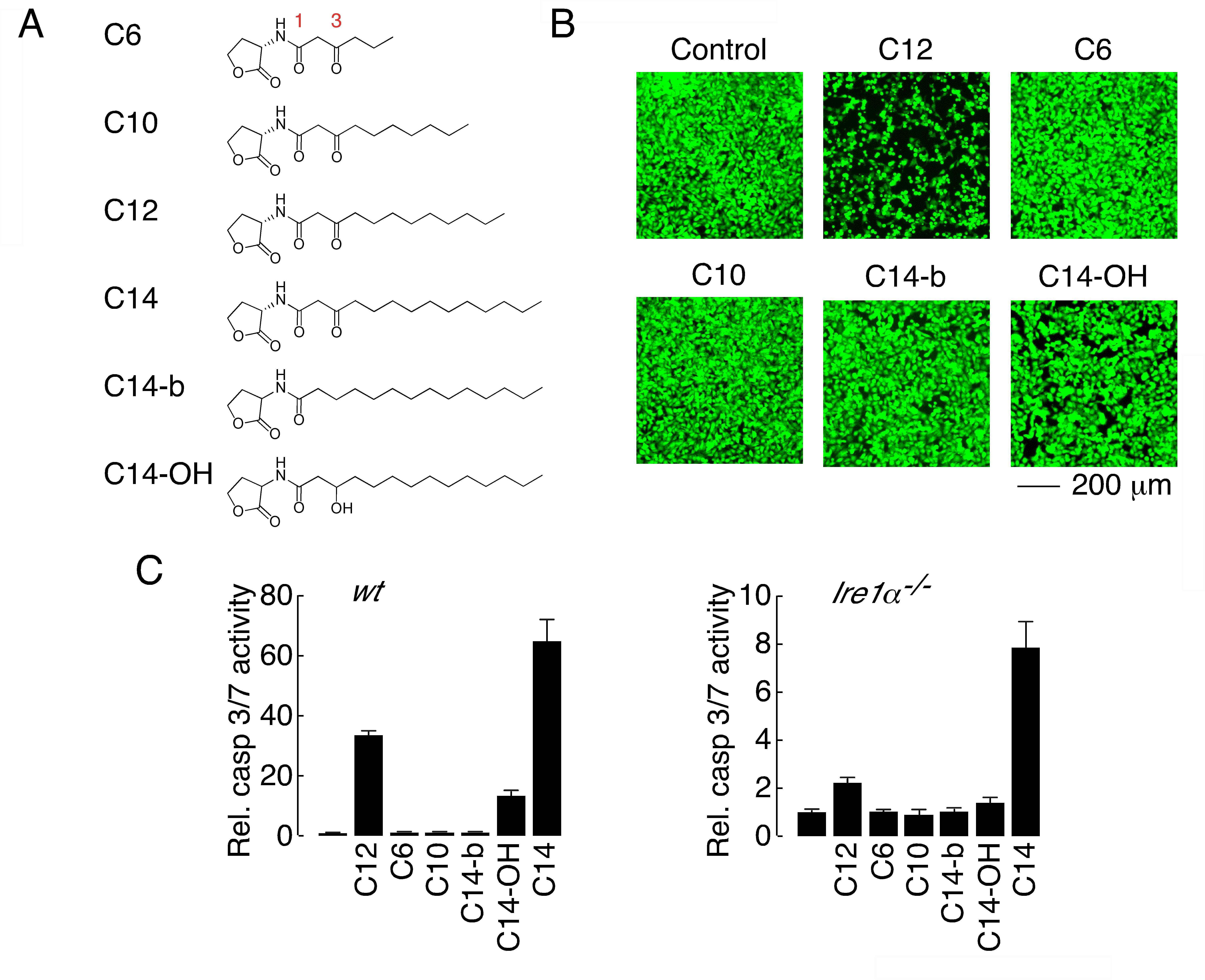

Supplement: Figure S3 — A. Structures of acyl homoserine lactones used in these studies: C6 (N-(ß-Ketocaproyl)-l-homoserine lactone); C10 (N-(3-Oxodecanoyl)-l-homoserine lactone); C12; C14; C14-b (N-Tetradecanoyl-dl-homoserine lactone); C14-OH (N-(3-Hydroxytetradecanoyl)-dl-homoserine lactone). Carbons at position 1 and 3 are highlighted in red (top). B. Representative images of calcein AM labelled wt MEFs treated with acyl homoserine lactones (4 h, 25 µM or 50 µM of racemic mixtures; see also Figs. 1 and 5). C. Caspase 3/7 activation in wt (left) and Ire1α−/− (right) MEFs after treatment with acyl homoserine lactones (4 h, 25 µM or 50 µM of racemic mixtures). No activation of caspases was observed when acyl chains were <12 carbon atoms in length. Substitutions at carbons one and three are required for caspase activation and 3-oxo- substitutions produce significantly greater activation of caspases than 3-hydroyx- substitutions. Ire1α−/− MEFs are protected from C12, C14 and C14-OH caspase activation relative to wt MEFs. (TIF) [file ppat.1003576.s003.tif]
