# Supplementary figures and images for: Correction: X-Box Binding Protein 1 (XBP1s) Is a Critical Determinant of Pseudomonas aeruginosa Homoserine Lactone-Mediated Apoptosis
Source: PLoS Pathog. 2016 May 18;12(5):e1005628. doi: 10.1371/journal.ppat.1005628 (PMC4871699; doi:10.1371/journal.ppat.1005628)

+STF-080310

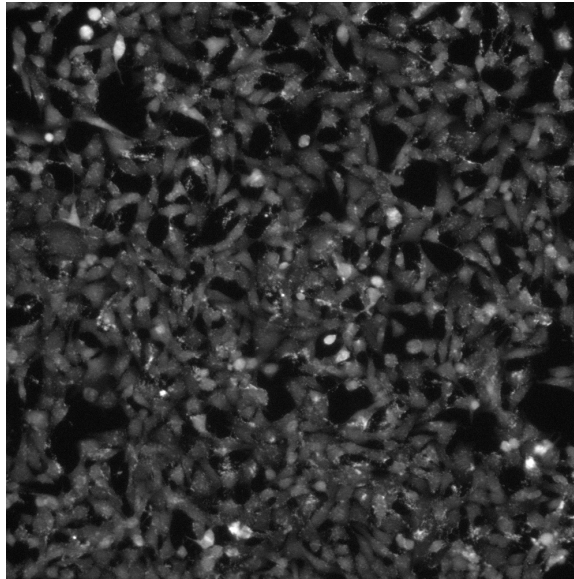

+C12

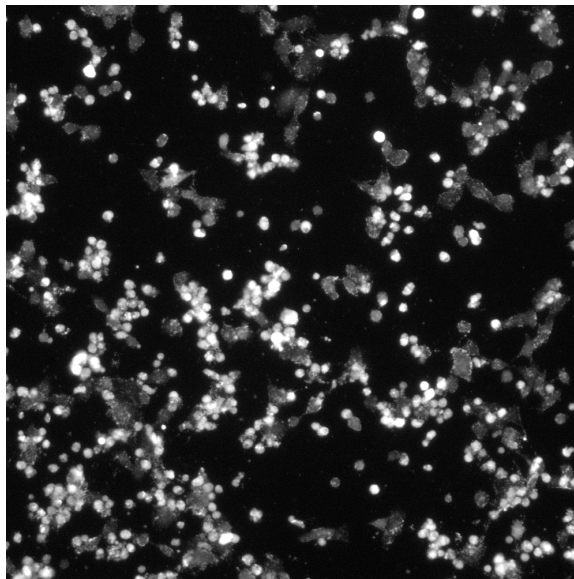

Supporting Information  
S1 File

Supplement: S1 Fig — Images were acquired as described in the Materials and Methods Section (using a Nikon TE2000 microscope equipped with a 16-bit Hamamatsu EM-CCD). To generate colorized images in Fig 2A, full-field images were binned (2×2), converted to 8-bit, colorized using a green lookup table, and brightness / contrast were adjusted to generate images that could be readily interpreted / visualized when presented in their final, published form. All image manipulations used Fiji and procedures were applied equally to all pixels in an image. To generate this Supporting Figure,.tif files were opened in Fiji, copied (using ‘Copy to System’ on an Apple computer), pasted into a Microsoft PowerPoint file, and saved as a PDF. (PDF) [file ppat.1005628.s001.pdf]
